# Extracellular microRNA and cognitive function in a prospective cohort of older men: The Veterans Affairs Normative Aging Study

**DOI:** 10.18632/aging.204268

**Published:** 2022-09-06

**Authors:** Nicole Comfort, Haotian Wu, Peter De Hoff, Aishwarya Vuppala, Pantel S. Vokonas, Avron Spiro, Marc Weisskopf, Brent A. Coull, Louise C. Laurent, Andrea A. Baccarelli, Joel Schwartz

**Affiliations:** 1 Department of Environmental Health Sciences, Columbia University Mailman School of Public Health, New York, NY 10032, USA; 2 Department of Obstetrics, Gynecology, and Reproductive Sciences, University of California San Diego, La Jolla, CA 92093, USA; 3 VA Normative Aging Study, VA Boston Healthcare System, Boston, MA 02130, USA; 4 Department of Medicine, Boston University School of Medicine, Boston, MA 02118, USA; 5 Massachusetts Veterans Epidemiology and Research Information Center, VA Boston Healthcare System, Boston, MA 02130, USA; 6 Department of Epidemiology, Boston University School of Public Health, Boston, MA 02118, USA; 7 Department of Psychiatry, Boston University School of Medicine, Boston, MA 02118, USA; 8 Department of Environmental Health, Harvard TH Chan School of Public Health, Boston, MA 02115, USA; 9 Department of Biostatistics, Harvard TH Chan School of Public Health, Boston, MA 02115, USA

**Keywords:** plasma, extracellular RNA, RNA-seq, microRNA, cognitive decline, cognitive impairment

## Abstract

Background: Aging-related cognitive decline is an early symptom of Alzheimer’s disease and other dementias, and on its own can have substantial consequences on an individual’s ability to perform important everyday functions. Despite increasing interest in the potential roles of extracellular microRNAs (miRNAs) in central nervous system (CNS) pathologies, there has been little research on extracellular miRNAs in early stages of cognitive decline. We leverage the longitudinal Normative Aging Study (NAS) cohort to investigate associations between plasma miRNAs and cognitive function among cognitively normal men.

Methods: This study includes data from up to 530 NAS participants (median age: 71.0 years) collected from 1996 to 2013, with a total of 1,331 person-visits (equal to 2,471 years of follow up). Global cognitive function was assessed using the Mini-Mental State Examination (MMSE). Plasma miRNAs were profiled using small RNA sequencing. Associations of expression of 381 miRNAs with current cognitive function and rate of change in cognitive function were assessed using linear regression (*N* = 457) and linear mixed models (*N* = 530), respectively.

Results: In adjusted models, levels of 2 plasma miRNAs were associated with higher MMSE scores (*p* < 0.05). Expression of 33 plasma miRNAs was associated with rate of change in MMSE scores over time (*p* < 0.05). Enriched KEGG pathways for miRNAs associated with concurrent MMSE and MMSE trajectory included Hippo signaling and extracellular matrix-receptor interactions. Gene targets of miRNAs associated with MMSE trajectory were additionally associated with prion diseases and fatty acid biosynthesis.

Conclusions: Circulating miRNAs were associated with both cross-sectional cognitive function and rate of change in cognitive function among cognitively normal men. Further research is needed to elucidate the potential functions of these miRNAs in the CNS and investigate relationships with other neurological outcomes.

## INTRODUCTION

As the U.S. population ages, there is growing concern about the loss of mental acuity associated with aging. At least 10% of individuals 65 years or older and 50% of those at least 85 years of age experience some impairment [1]. These declines can have substantial consequences on an individual’s ability to perform important everyday functions [2, 3]. Non-clinical aging-related cognitive decline is also an early symptom of Alzheimer’s disease (AD) and related dementias [4, 5]. The exact mechanisms of initiation of cognitive decline remain unclear which has hampered progress in identifying risk factors, treatments, and implementing targeted prevention. Elucidating the biological processes involved in aging-related cognitive decline may significantly advance our ability to not only detect but also to ultimately prevent or mitigate aging-related cognitive impairments and clinical dementia such as AD.

Circulating extracellular RNAs (exRNAs) including microRNAs (miRNAs) have recently gained attention for their possible association with neurodegenerative disease pathogenesis and progression [6–8]. Altered levels of extracellular miRNAs have been detected among patients with mild cognitive impairment and AD [9–15], and a recent study has investigated plasma miRNAs as potential biomarkers to predict progression from mild cognitive impairment to overt dementia [16]. However, to our knowledge, no studies have investigated associations between extracellular miRNAs and cognitive function among pre-clinical (i.e., cognitively “normal”) older individuals. Understanding the functions of miRNAs in the earliest stages of cognitive decline will expand our knowledge on the biology of prodromal AD and the roles of circulating miRNAs in neurodegenerative diseases and could result in identification of therapeutic targets to guide drug development [17].

In this work, our aim was to test the hypothesis that circulating plasma miRNAs are associated with current cognitive function and rate of cognitive decline in the Normative Aging Study (NAS), a prospective cohort of older men. The primary objective of this study was to examine the association of plasma miRNA expression, quantified using small RNA sequencing (RNA-seq), with cognitive function and rate of cognitive decline in this cohort. Our secondary objective was to explore the biological pathways associated with the gene targets of any miRNAs associated with cognitive function or rate of cognitive decline. We found that (i) 2 plasma miRNAs are cross-sectionally associated with higher Mini-Mental State Examination (MMSE) scores; (ii) 33 plasma miRNAs are associated with trajectory of MMSE scores over time; and (iii) these miRNAs are associated with prion diseases, fatty acid biosynthesis, Hippo signaling, and extracellular matrix (ECM)-receptor interactions.

## RESULTS

### Descriptive statistics of study participants

**Figure 1:**
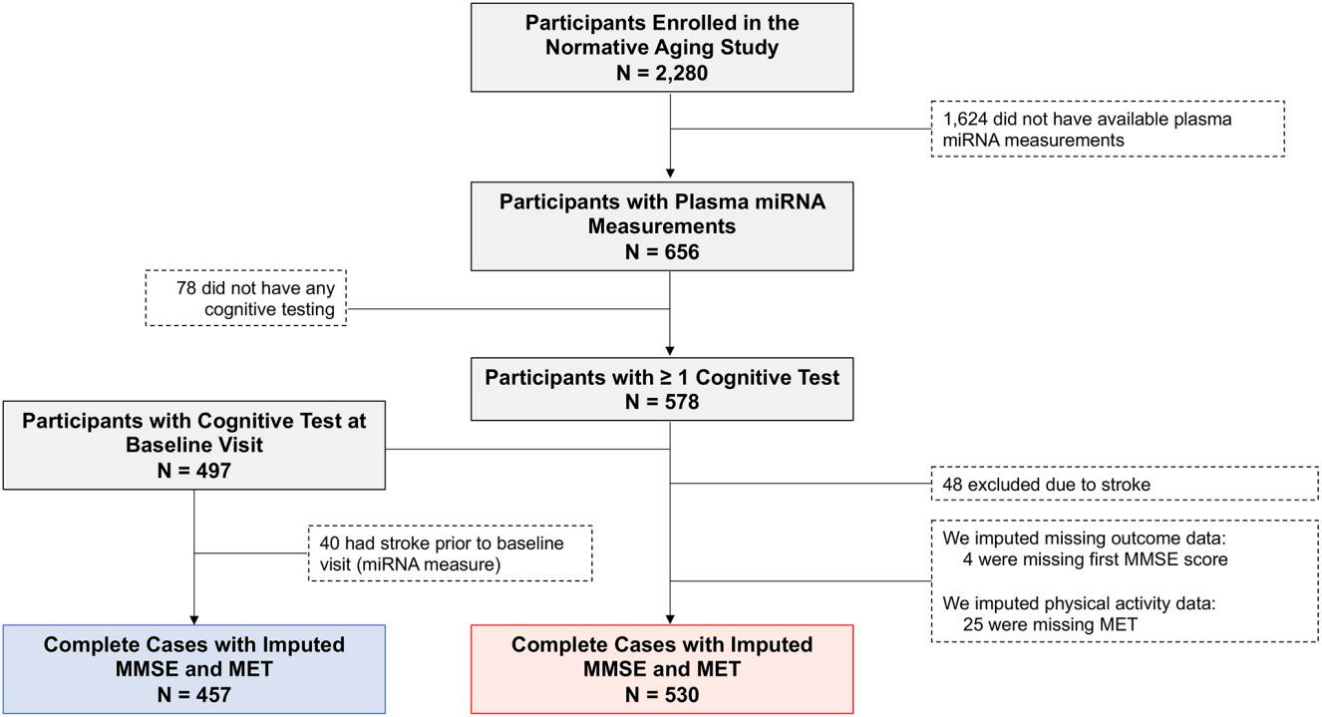
**Flow diagram depicting eligible and non-eligible Normative Aging Study (NAS) participants.*** N* = 530 subjects (red box) were included in the analysis of MMSE trajectory. *N* = 457 of these subjects (blue box) had MMSE assessed at the same visit as the plasma miRNA measure and were included in an additional cross-sectional analysis. Abbreviations: MMSE: Mini-Mental State Examination; MET: Metabolic equivalent of task, measured in hours/week. One MET is defined as the energy expenditure for sitting quietly, which, for the average adult, approximates 3.5 mL of oxygen uptake per kilogram of body weight per minute (1.2 kcal/min for a 70-kg individual).

A total of 530 men with 1,331 person-visits (equivalent to 2,471 years of follow up) met the inclusion criteria for this study (Figure 1). Table 1 summarizes the baseline characteristics of the cohort. The mean ± standard deviation (SD) age of the sample at baseline was 72.0 ± 6.8 years (median: 71.0 years; range: 55–94 years) and the mean ± SD maximum level of education was 14.9 ± 2.9 years, corresponding to at least some college education. Most of the men were white (96.8%), not heavy drinkers (82.6%), and were current or former smokers (70.2%). Physical activity exhibited a skewed distribution, with a median of 7.75 metabolic equivalent of task hours (MET-hrs) per week. Most men were not diabetic (82.1%) but were diagnosed with hypertension (71.5%). The median number of visits was 2 (range: 1–6), while the median years of follow up was 6.0 years (range: 0–14 years, interquartile range: 0–9). The first cognitive assessment in this study (at or after baseline, whichever comes first) was the first ever cognitive assessment for 38 participants (7.2%). Within this sample, 457 men had MMSE assessed at the baseline visit (i.e., the same visit as the plasma miRNA measure). Descriptive statistics for MMSE scores across cognitive assessments are shown in Table 2.

**Table 1:** Baseline characteristics of Normative Aging Study (NAS) participants (*N* = 530).

	**Included NAS Participants**
	**(*N* = 530)**
**Age (years)**	
Mean (SD)	72.0 (6.8)
Median (Min, Max)	71.0 (55.0, 94.0)
**Race/Ethnicity**	
White	513 (96.8%)
Black	12 (2.3%)
Hispanic	4 (0.8%)
Missing	1 (0.2%)
**Max Education (years)**	
Mean (SD)	14.9 (2.9)
Median (Min, Max)	14.0 (6.0, 29.0)
**Alcohol Consumption (drinks/day)**	
<2	438 (82.6%)
≥2	92 (17.4%)
**Smoking Status**	
Never	158 (29.8%)
Current/Former	372 (70.2%)
**Physical Activity (MET-hrs/week)**	
Mean (SD)	14.3 (17.8)
Median (Min, Max)	7.75 (0.25, 138.17)
1st quartile, 3rd quartile	3.09, 19.62
Missing	29 (5.5%)
**Diabetes**	
No	435 (82.1%)
Yes	95 (17.9%)
**Hypertension**	
No	151 (28.5%)
Yes	379 (71.5%)
**Number of Visits**	
Median (Min, Max)	2 (1, 6)
IQR	1-3
**Years of Total Follow Up**	
Median (Min, Max)	6 (0, 14)
IQR	0−9

These men had at least one cognitive assessment on or after the baseline visit, defined as the visit when blood was drawn for plasma miRNA analysis. Abbreviations: IQR: Interquartile range; SD: Standard deviation; MET: Metabolic equivalent of task, measured in hours (hrs) per week. One MET is defined as the energy expenditure for sitting quietly, which, for the average adult, approximates 3.5 mL of oxygen uptake per kilogram of body weight per minute (1.2 kcal/min for a 70-kg individual).

**Table 2:** Mini-Mental State Examination (MMSE) scores.

**Cognitive assessment**	***N***	**Age**	**MMSE**		
		**Mean age (Min, Max)**	**Mean (SD)**	**Min, Max**	**Missing (no., %)**
1	530^*^	72.58 (55, 94)	26.57 (1.97)	15, 29	4 (0.75%)
2	370	74.79 (59, 94)	26.66 (1.94)	13, 29	8 (2.16%)
3	268	77.35 (64, 97)	26.73 (1.81)	20, 29	27 (10.07%)
4	138	79.01 (67, 96)	26.60 (1.96)	18, 29	6 (4.35%)
5	24	82.21 (75, 93)	26.83 (2.57)	17, 29	0 (0%)
6	1	80	23 (NA)	NA	0 (0%)

^*^For *N* = 457, the first visit occurred at the baseline visit, i.e., the same visit when blood was drawn for the plasma miRNA measure. The remaining 73 participants had their first MMSE measure occur after the baseline plasma assessment. Abbreviations: SD: Standard deviation; NA: Not available.

### Descriptive statistics of plasma miRNAs

A total of 381 plasma miRNAs passed quality control checks and were detected in ≥ 70% of participants. Descriptive statistics of the normalized, batch-corrected reads (in counts per million) of these 381 miRNAs are presented in Supplementary Table 1. Among all miRNA pairs, 42.0% were significantly correlated (Spearman correlation Bonferroni-corrected *p* < 0.05), and most significantly correlated miRNAs (57.7%) were positively correlated (Supplementary Figure 1).

### Circulating miRNAs and cross-sectional cognitive function

**Figure 2:**
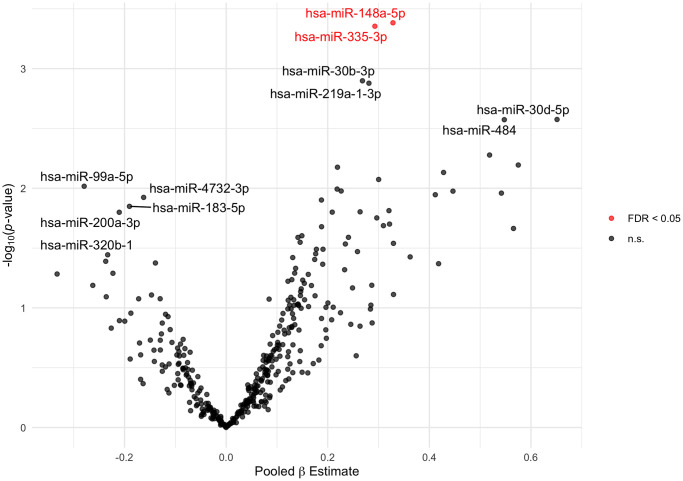
**Volcano plot of plasma miRNAs associated with baseline MMSE score.** Estimates (pooled from ten imputed datasets) from linear models adjusted for age, education, alcohol consumption, and smoking status. The 5 and 6 most statistically significant extracellular miRNAs with negative and positive beta estimates, respectively, are labeled. The 2 extracellular miRNAs significant at FDR *q*-value < 0.05 are plotted in red. Abbreviations: FDR: False Discovery Rate; MMSE: Mini-Mental State Examination; n.s.: not significant.

At baseline (i.e., the study visit when blood was drawn for the plasma miRNA measure), relative abundance of hsa-miR-148a-5p and hsa-miR-335-3p was positively associated with MMSE in our multivariable-adjusted linear model (Figure 2). A doubling in relative abundance of hsa-miR-148a-5p was associated with a 0.33-point higher MMSE score (95% confidence interval [CI]: 0.15 to 0.51; False discovery rate [FDR] *q*-value < 0.05) and a doubling in relative abundance of hsa-miR-335-3p was associated with a 0.29-point higher MMSE score (95% CI: 0.13 to 0.46; FDR *q*-value < 0.05) (Supplementary Table 2). For comparison, an additional year of age was associated with a 0.06-point lower MMSE score. Neither hsa-miR-148a-5p or hsa-miR-335-3p was correlated with age or education, and the distribution of the miRNA’s relative abundance did not differ by smoking status (Supplementary Figures 2, 3). Our results were materially unchanged in sensitivity analyses (Supplementary Figure 4).

We next performed an enrichment analysis to identify biological pathways enriched with genes targeted by either hsa-miR-148a-5p or hsa-miR-335-3p (506 and 430 experimentally validated genes, respectively). Experimentally validated gene targets were identified using TarBase v7.0 [18]. The KEGG (Kyoto Encyclopedia of Genes and Genomes) functional enrichment analysis revealed that these miRNA targets are associated with the Hippo signaling pathway, ECM-receptor interactions, and galactose metabolism (FDR *q*-values: <0.0001, <0.0001, 0.0076, respectively) (Table 3). To further explore the biological functions of these miRNAs, we performed pathway enrichment analysis using the predicted miRNA target genes, identified using the microT-CDS v.5 [19] database in DIANA-miRPath (625 genes for hsa-miR-148a-5p; 2,984 genes for hsa-miR-335-3p). These results are shown in Supplementary Figure 5. Again, various pathways targeted by these miRNAs were identified, including pathways associated with transforming growth factor beta (TGF-β) and Hippo signaling, ECM-receptor interactions, and glioma (FDR *q*-values of <0.00001, 0.0058, 0.0065, and 0.0002, respectively).

**Table 3:** Results of KEGG pathway enrichment analysis of experimentally validated gene targets of plasma miRNAs cross-sectionally associated with MMSE: hsa-miR-148a-5p and hsa-miR-335-3p.

**KEGG pathway**	**FDR *q*-value**	**# Genes**	**Genes**
Hippo signaling pathway	5.64E-11	11	*GSK3B, YWHAH, YAP1, SMAD3, WWTR1, YMHAQ, MMP5, FRMD6, TEAD1, LATS1, SERPINE1*
ECM-receptor interaction	1.23E-06	6	*ITGB1, THBS1, THBS2, COL3A1, COL1A2, SDC4*
Galactose metabolism	0.0076	2	*UGP2, GLA*
Colorectal cancer	0.0566	7	
Pantothenate and CoA biosynthesis	0.1089	2	
Shigellosis	0.1097	6	
mRNA surveillance pathway	0.1318	7	
ErbB signaling pathway	0.1671	10	
Endometrial cancer	0.1699	6	

These two miRNAs are associated with continuous MMSE score at baseline. Experimentally validated gene targets were identified using TarBase v7.0. Enriched gene targets are listed for pathways meeting statistical significance. Abbreviations: ECM: Extracellular matrix; CoA: Coenzyme A; MMSE: Mini-Mental State Examination.

### Circulating miRNAs and rate of cognitive decline

**Figure 3:**
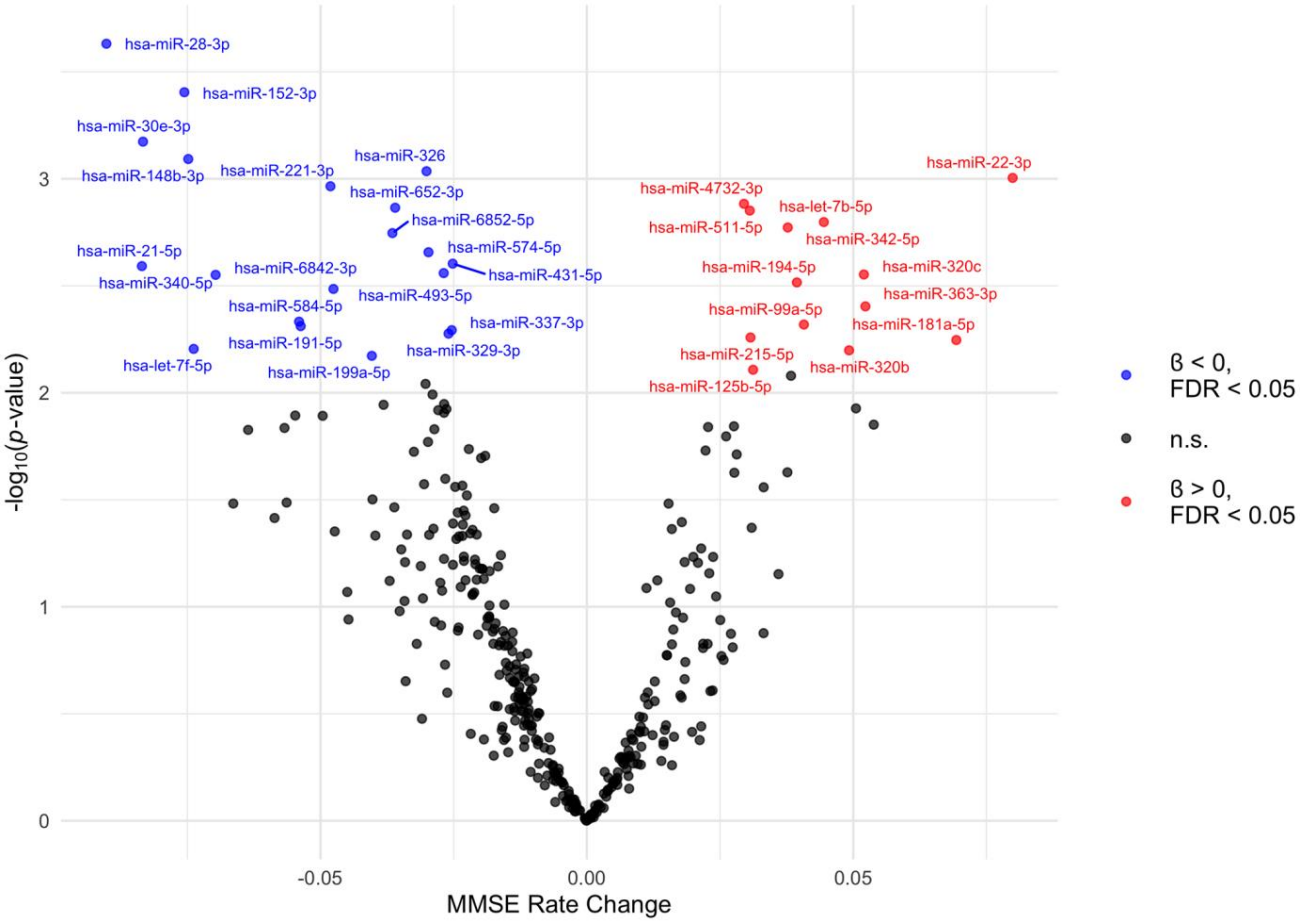
**Volcano plot of plasma miRNAs associated with trajectory of MMSE scores.** The MMSE rate change (x axis) is the equivalent of the regression coefficient of the interaction term in the linear mixed model. These beta estimates, pooled from ten imputed datasets, are from linear mixed models adjusted for age, education, alcohol consumption, smoking status, and follow up time. The 33 extracellular miRNAs that interacted with follow up time significant at FDR *q*-value < 0.05 are labelled. MiRNAs with positive and negative interaction terms are plotted in red and blue, respectively. Abbreviations: FDR: False Discovery Rate; MMSE: Mini-Mental State Examination; n.s.: not significant.

We then investigated the potential association between baseline plasma miRNA abundance and longitudinal changes in cognition. As expected, MMSE scores displayed a slight overall decline with age. In bivariate analyses, MMSE was significantly associated with age, with a 0.06-point decrease in MMSE score per year (95% CI: −0.07 to −0.04; *p* < 0.0001). In our linear mixed models assessing repeated measures of MMSE, we observed significant interactions between follow up time and 33 plasma miRNAs on MMSE, which indicates that baseline levels of each of these 33 unique miRNAs were associated with the trajectory of MMSE score over time (Figure 3, Table 4). We identified 13 miRNAs with positive interaction terms and 20 miRNAs with negative interaction terms, meaning these miRNAs are associated with an increase and decrease in global cognitive function, respectively. Among participants that experienced a progressive decline in MMSE (virtually all participants), these miRNAs are associated with slower and faster declines in global cognitive function, respectively. The full results of this analysis (for all miRNAs tested) are included in Supplementary Table 3.

**Table 4:** Plasma miRNAs (33) associated with trajectories of MMSE scores over follow up time (*N* = 530).

**miRNA**	**Pooled β Value**	**95% Confidence interval**	**FDR *q*-value**	**Sign of interaction term**
hsa-miR-28-3p	−0.0902	−0.14, −0.04	0.0287	−
hsa-miR-152-3p	−0.0756	−0.12, −0.03	0.0287	−
hsa-miR-30e-3p	−0.0833	−0.13, −0.04	0.0287	−
hsa-miR-148b-3p	−0.0748	−0.12, −0.03	0.0287	−
hsa-miR-326	−0.0301	−0.05, −0.01	0.0287	−
hsa-miR-22-3p	0.0799	0.03, 0.13	0.0287	+
hsa-miR-221-3p	−0.0481	−0.08, −0.02	0.0287	−
hsa-miR-4732-3p	0.0295	0.01, 0.05	0.0287	+
hsa-miR-652-3p	−0.0360	−0.06, −0.01	0.0287	−
hsa-miR-511-5p	0.0306	0.01, 0.05	0.0287	+
hsa-let-7b-5p	0.0445	0.02, 0.07	0.0287	+
hsa-miR-342-5p	0.0377	0.01, 0.06	0.0287	+
hsa-miR-6852-5p	−0.0365	−0.06, −0.01	0.0287	−
hsa-miR-574-5p	−0.0297	−0.05, −0.01	0.0308	−
hsa-miR-431-5p	−0.0252	−0.04, −0.01	0.0308	−
hsa-miR-21-5p	−0.0835	−0.14, −0.03	0.0308	−
hsa-miR-493-5p	−0.0269	−0.04, −0.01	0.0308	−
hsa-miR-320c	0.0520	0.02, 0.09	0.0308	+
hsa-miR-340-5p	−0.0697	−0.12, −0.02	0.0308	−
hsa-miR-194-5p	0.0394	0.01, 0.07	0.0317	+
hsa-miR-6842-3p	−0.0476	−0.08, −0.02	0.0324	−
hsa-miR-363-3p	0.0523	0.02, 0.09	0.0373	+
hsa-miR-584-5p	−0.0540	−0.09, −0.02	0.0406	−
hsa-miR-99a-5p	0.0407	0.01, 0.07	0.0406	+
hsa-miR-191-5p	−0.0537	−0.09, −0.02	0.0406	−
hsa-miR-337-3p	−0.0254	−0.04, −0.01	0.0406	−
hsa-miR-329-3p	−0.0260	−0.04, −0.01	0.0406	−
hsa-miR-215-5p	0.0307	0.01, 0.05	0.0406	+
hsa-miR-181a-5p	0.0693	0.02, 0.12	0.0406	+
hsa-let-7f-5p	−0.0738	−0.13, −0.02	0.0425	−
hsa-miR-320b	0.0492	0.01, 0.08	0.0425	+
hsa-miR-199a-5p	−0.0404	−0.07, −0.01	0.0437	−
hsa-miR-125b-5p	0.0312	0.01, 0.05	0.0492	+

The beta estimate represents the beta estimate (pooled from ten imputed datasets) for the interaction of miRNA and follow up time from linear mixed models adjusted for follow up time since baseline and baseline age, education, alcohol consumption, and smoking status. MiRNAs are ordered by ascending False Discovery Rate (FDR). Shown here are the 33 miRNAs significant at FDR < 0.05 (also colored and labelled in Figure 3). Full results from this analysis are included in Supplementary Table 3. MMSE: Mini-Mental State Examination.

**Figure 4:**
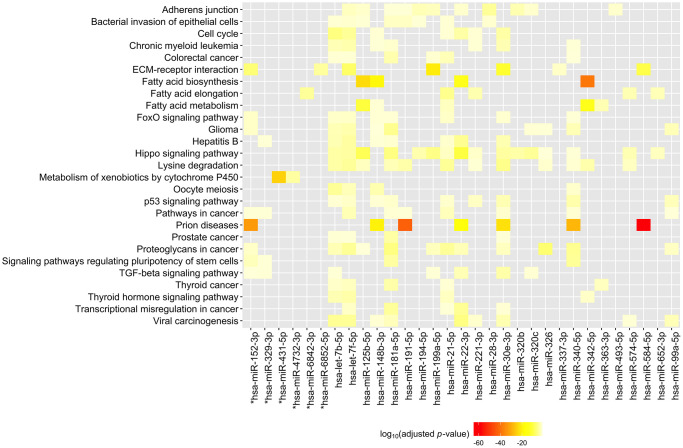
**Heatmap of KEGG pathways targeted by 33 plasma miRNAs associated with trajectory of MMSE scores.** MiRNA gene targets were identified using TarBase v7.0. Inset shows the log_10_(FDR *q*-value). MMSE: Mini-Mental State Examination. ^*^For these miRNAs, predicted gene targets identified using microT-CDS v.5 were used as input to the KEGG analysis.

We again used DIANA-miRPath to perform functional enrichment analysis of these 33 miRNAs to clarify their possible biological roles. In this analysis, we used experimentally validated as well as predicted miRNA gene targets as inputs to the analyses, using the predicted gene targets for 8 miRNAs which had no experimentally validated mRNA targets. A heatmap of the KEGG pathways significantly enriched with gene targets of these miRNAs is shown in Figure 4. Full results of the KEGG analysis can be found in Supplementary Table 4. The KEGG functional enrichment analysis of the 33 significant miRNAs revealed that they are associated with: fatty acid biosynthesis (*p* < 0.00001, 3 gene targets) and fatty acid metabolism (*p* < 0.00001, 13 gene targets), prion diseases (*p* < 0.00001, 9 gene targets) and glioma (*p* < 0.00001, 41 gene targets), as well as ECM-receptor interactions (*p* < 0.00001, 30 gene targets), the Hippo signaling pathway (*p* < 0.00001, 89 gene targets), and the TGF-β signaling pathway (*p* = 0.0018, 47 gene targets), which were also associated with miRNAs hsa-miR-148a-5p and hsa-miR-335-3p, indicating the importance of these pathways in both current cognitive function and rate of change in cognitive function.

Our results did not meaningfully change in sensitivity analyses adjusting for additional covariates and when restricting the analysis to white participants (Supplementary Figure 6). To control for loss to follow up, we computed inverse probability of censoring weights and applied these in weighted linear mixed models using participants with no missing outcome data (*N* = 526). Though the nominal *p*-values from these weighted models broadly agreed with those of the analogous unweighted models (*R^2^* = 0.44) (Supplementary Figure 7), the *p*-values from the weighted models were lower than the unweighted models, resulting in an even greater number of miRNAs meeting statistical significance (82 miRNAs with FDR *q*-value < 0.05). The top 5 KEGG pathways targeted by these 82 miRNAs were prion diseases, fatty acid biosynthesis, ECM-receptor interaction, fatty acid metabolism, and glioma (*p* < 0.00001) (Supplementary Table 5). Among these 82 significant miRNAs, 30 were also significant in our original results (out of 33), indicating there was high overlap between the miRNAs identified by unweighted linear mixed models using imputed outcome data and miRNAs identified by linear mixed models using complete cases and controlling for selection bias by applying inverse probability of censoring weights. Furthermore, significant KEGG pathways of these 30 miRNAs included fatty acid biosynthesis, prion diseases, Hippo signaling, ECM-receptor interactions, glioma (*p* < 0.00001), and TGF-β signaling (*p* = 0.0018). Thus, our results are reasonably robust to missing data and selection bias.

## DISCUSSION

In a cohort of older men from Massachusetts, we investigated associations between plasma miRNAs and global cognition and rate of global cognitive decline measured by the MMSE. Among 381 plasma miRNAs detectable in at least 70% of samples, expression of 2 miRNAs were associated with baseline (i.e., cross-sectional) MMSE scores (FDR *q*-value = 0.048) and levels of 33 miRNAs were associated with rate of change in MMSE over time (FDR *q*-value < 0.05), suggesting that plasma miRNA levels are associated with both the level of and change in global cognition among older men. We explored the potential regulatory targets of significant plasma miRNAs using KEGG enrichment analysis. The genes targeted by these miRNAs are mainly associated with prion diseases and fatty acid biosynthesis as well as with Hippo signaling, ECM-receptor interactions, and TGF-β signaling pathways. These latter three results were consistent for both the cross-sectional and longitudinal analyses. These pathways provide insight into the possible contribution of these miRNAs to aging-related cognitive decline.

For instance, the Hippo signaling pathway is a highly conserved signaling mechanism that chiefly manages cell survival, cell proliferation, and apoptosis, though it also plays a critical role in the development of the nervous system at multiple stages, from neural stem cell proliferation and migration to neuronal differentiation, synaptogenesis, and neuronal death [20, 21]. It plays important roles in balancing regulated neuronal death such that hypo- as well as hyper-activation of this pathway can result in pathological conditions such as glioma and neurodegeneration, respectively [20, 22–24]. Thus, the appearance of target genes enriched in “glioma” in our results could also indicate aberrant Hippo signaling. Hippo signaling is also activated in amyloid-β-mediated neurodegeneration [25], suggesting that inhibitors of Hippo signaling could be a therapeutic target for prevention of cognitive decline.

Hippo signaling also plays a critical role in the formation and maintenance of synapses, dendritic arborization, and axon guidance and elongation which has implications for various neurodegenerative diseases such as AD and related dementias [26]. Inappropriate development and maintenance of synapses can also alter neural circuit formation which has known impacts on cognitive decline and learning/memory [27, 28]. ECM receptors and their ligands also play key roles in axonal projections, dendrite structure, synapse formation, function, and maintenance, and synaptic plasticity. Consequently, disruptions of integrin subunits can result in learning and memory impairments [29].

KEGG pathway analyses of the plasma extracellular miRNA targets also identified TGF-β signaling as an enriched pathway. TGF-βs play important neuroprotective functions in response to injury. TGF-β signaling increases with age and is induced by both acute and chronic brain injury including neurodegeneration and AD [30, 31]. It has been suggested that in AD, reduced trophic support to neurons combined with age-dependent increases in cellular stress results in chronic injury leading to neuronal death. Indeed, in a mouse model of AD, a reduction of TGF-β signaling in neurons resulted in age-dependent neurodegeneration and promoted AD-like pathology [32]. Evidence of the role of TGF-β signaling in neurodegeneration and AD pathology establishes this signaling pathway as a potential therapeutic target for these disorders [33–35].

Lastly, gene targets of plasma miRNAs identified in the repeated measures analyses were enriched in pathways related to fatty acid biosynthesis and prion diseases. Increasing evidence suggests that abnormal fatty acid metabolism is associated with cognitive dysfunction [36] and neurological disorders such as AD [37–39]. Prion diseases are transmissible neurodegenerative disorders characterized by the presence and propagation of misfolded prion proteins. AD also involves propagation and aggregation of misfolded proteins, and a growing number of studies have suggested that extracellular vesicles (EVs, a source of exRNA) can contribute to prion-like propagation of amyloid deposits and neurofibrillary tangles in AD [40]. On the other hand, normal functioning prion protein is involved in processes including synaptic function and transmission, hippocampal synaptic functioning, metal transport, and copper homeostasis [41]. Furthermore, it has been demonstrated that in AD, prion protein on the membranes of EVs serves a neuroprotective role by binding to amyloid-β oligomers and converting these toxic amyloid-β entities into non-toxic species [42]. Thus, there may be a neuroprotective role of prion protein which is void upon conversion to the misfolded, pathological form.

Taken together, our results suggest that various plasma extracellular miRNAs are associated with global cognitive function among cognitively “normal” men. If they were to play a causal role in cognitive decline, it would likely be through pathways that regulate synaptic plasticity, cell death, the response to injury, and energy homeostasis. Extracellular miRNAs may also contribute to greater rates of cognitive decline possibly through involvement in the prion-like propagation of AD lesions or in hindering their neuroprotective role of curbing AD-like pathology. We observed overlap between the miRNAs identified in our study and those detected in previous studies assessing extracellular miRNA profiles in relation to cognitive function and neuropathological features among AD patients. For instance, a recent study observed a strong inverse relationship between plasma levels of the inflammatory miRNA miR-21-5p and MMSE of patients with AD compared to healthy controls [15]. In another recent study by Wiedrick and colleagues assessing the utility of extracellular miRNAs in human cerebrospinal fluid (CSF) of AD patients and controls as biomarkers for AD [9, 43], the miRNAs miR-28-3p, miR-125b-5p, and miR-584-5p were among the candidate disease biomarkers identified and were correlated with MMSE scores [9]. Each of these miRNAs were also associated with MMSE trajectory in our study. Our results also overlapped with the results of another study by Burgos and colleagues comparing the extracellular miRNA profiles in CSF and serum of AD patients to healthy controls [44]; these investigators found 41 miRNAs differentially expressed in CSF and 20 miRNAs differentially expressed in serum of AD patients compared to controls. Among these miRNAs, four were also identified in our study (miR-181a-5p, miR-326, miR-329-3p, and miR-21-5p) to be associated with rate of change in cognition. The Burgos et al. study also observed that CSF levels of the miRNA miR-181a-5p negatively correlated with disease progression (assessed by Braak stages and neurofibrillary tangle score). Thus, extracellular miRNAs detectable in the periphery can reflect cellular changes associated with neuropathology, but their definitive role in disease progression remains to be elucidated. Differences in the miRNAs identified in our study and other studies can be attributed to differences in the biofluid matrix, its collection and storage, participant characteristics, and methods of RNA isolation and miRNA expression profiling.

Our results are consistent with the findings of a recent study by Nie and colleagues that sequenced plasma EV-encapsulated miRNAs of patients with AD, Parkinson’s disease, and healthy controls [45]. Although only one miRNA we identified was also detected as differentially expressed among AD patients in the Nie et al. study (hsa-let-7b-5p), as we demonstrated in the results of our linear regression and linear mixed models, their cross-sectional study also reported that the KEGG pathways of the miRNAs they found differentially regulated in AD patients included fatty acid biosynthesis, Hippo signaling, ECM-receptor interactions, and TGF-β signaling. Furthermore, two out of the five AD patients in the Nie et al. study had MMSE scores within the range of the NAS participants’ scores in this analysis. Another study that assessed the levels of circulating extracellular miRNAs in plasma of AD patients and healthy controls found that miR-191-5p, which was associated with MMSE trajectory in our analysis, was down-regulated among AD patients and pathways identified in ingenuity pathway analysis that were significantly enriched with signature miRNA targets included axonal guidance signaling [46].

This study is subject to limitations. First, our results may be subject to selection bias due to loss to follow up, as subjects experiencing cognitive impairments would likely have dropped out of the study. However, 30 of the 33 miRNAs identified by our repeated measures analyses remained statistically significant in a sensitivity analysis using inverse probability of censoring weights, and the top enriched KEGG pathways were not affected. Another limitation is that we ran single-miRNA analyses and thus we are unable to evaluate joint (e.g., synergistic or antagonistic) effects of a suite of miRNAs acting in tandem [47]. Also, compared to other cognitive tests, the MMSE is relatively poor in identifying certain cases of cognitive impairment such as frontotemporal dementia [48]. Thus, additional screening tools such as the Cambridge Neuropsychological Test Automated Battery (CANTAB) and the Addenbrooke’s Cognitive Examination – III should be used alongside the MMSE in future studies of cognitive decline in healthy, aging populations [48, 49]. Although we analyzed repeated measures of cognitive function, this study is limited to a single plasma miRNA measure, and thus it is unknown how the abundance of these circulating miRNAs may fluctuate over time. Lastly, these results are based on a cohort of older, mostly white men and may be generalizable only to populations with similar characteristics [50]. However, the results of our KEGG enrichment analyses are in close agreement with another study that examined the plasma EV-miRNA profile of patients with AD that were mostly female (4 out of 5) and healthy controls that were younger and contained an equal balance of males and females [45]. Nonetheless, our findings should be replicated in larger cohorts and studied concomitantly with neuroimaging and other biological measurements used in diagnosis of suspected dementia.

A major strength of this study is that it used repeated measures of cognition (up to six assessments) spanning a period of up to 14 years (median follow up: 6 years), allowing us to detect changes in cognitive performance over time. Future studies should examine longitudinal changes in plasma exRNA for prediction of later clinical diagnosis of neurodegenerative diseases such as AD. Our results were robust to sensitivity analyses we performed which included outlier removal, adjusting for additional covariates, restricting the analysis to white participants, and controlling for selection bias. Another strength of this study is that we employed small RNA-seq, a novel deep-sequencing approach that provides more precise miRNA measurements than alternative methods such as microarrays [51, 52]. RNA-seq has many advantages over microarrays which cover only a defined set of transcripts, exhibit high background levels due to cross-hybridization, and embody a limited dynamic range [53]. In a future study, we will validate select extracellular miRNAs using quantitative real-time PCR (qRT-PCR). Experimental studies could then confirm the functional role of these miRNAs in the central nervous system (CNS). Also, in an effort to identify potential functional biomarkers that may also serve as therapeutic targets, future studies should assess extracellular miRNAs from peripheral biofluids alongside matched brain tissue samples to determine the degree to which changes in peripheral miRNAs reflect changes in CNS miRNA expression and concurrent neuropathologic changes.

In conclusion, while studies have investigated extracellular miRNA profiles and cognitive function and/or disease progression among patients with mild cognitive impairment and AD, to our knowledge, no study has investigated the association between circulating miRNAs and age-related cognitive impairment among cognitively “normal” individuals not diagnosed with dementia. In this study, we detected associations between plasma extracellular miRNAs and cross-sectional MMSE and with rate of change in MMSE over time. Biological pathways enriched among identified miRNAs included the Hippo signaling pathway and ECM-receptor interactions. Additionally, fatty acid biosynthesis and prion diseases were the main biological pathways targeted by miRNAs associated with trajectories of cognitive function. These findings warrant replication in experimental studies and follow up in larger cohorts with repeated measures from subjects that have been characterized in terms of cognitive and imaging data, other biomarkers for cognitive decline (e.g., CSF tau levels), and AD risk factors (e.g., apolipoprotein-E gene [*APOE*] status). If these circulating miRNAs are causally associated with cognitive decline, this would have implications for drug development and therapeutic monitoring of neurodegenerative disorders such as cognitive impairment. The investigation and verification of these circulating plasma miRNAs as potential mechanistic biomarkers of cognitive decline warrants further investigation. Additional research is needed to identify the biological pathways influenced by expression of these extracellular miRNAs, investigate relationships with CNS miRNA expression, and examine the potential impacts of circulating miRNAs on other neurological outcomes.

## MATERIALS AND METHODS

### Study sample

The U.S. Department of Veterans Affairs (VA) Normative Aging Study (NAS) is an ongoing longitudinal cohort study of aging that was established in 1963 with men from the Greater Boston, Massachusetts area. Details on the NAS cohort have been reported previously [54]. Briefly, 2,280 men 21-81 years old and free of chronic medical conditions were recruited to undergo an in-person examination every 3-5 years. At each visit, participants underwent a physical examination and laboratory tests and provided information on medical history, lifestyle, and demographic factors.

Starting in 1993, participants have been asked to complete a brief battery of cognitive tests [55, 56]. Collection of blood samples for molecular analysis, such as plasma miRNA profiling, began in January 1996. The present analysis is limited to participants for whom we were able to obtain plasma miRNA data and who also completed at least one cognitive assessment at the visit when plasma was collected for miRNA analysis, considered the participant’s baseline visit for this study, or after this visit. This ensures that we did not include cognitive test data obtained before the miRNA measure in our analyses. Out of 656 participants with plasma miRNA data, 578 (88.1%) underwent cognitive testing at this baseline visit or thereafter.

Forty-eight participants (8.3%) who experienced a stroke before their first cognitive assessment (at the baseline visit or after, whichever came first) were excluded from the study, resulting in 530 individuals included in the analysis (Figure 1). 457 (86.2%) of these men completed cognitive testing at their baseline visit, while 73 (13.8%) participants underwent cognitive testing at a later visit. Compared to those who were included in the analysis, men excluded due to stroke were older (mean age ± SD of 77.2 ± 7.5 vs. 72.0 ± 6.8 years, respectively, *p* < 0.0001), but they did not differ with respect to race/ethnicity, education level, alcohol consumption, smoking status, physical activity, diabetes and hypertension status, and baseline global cognitive function (Supplementary Table 6). Over the course of cognitive follow up, participants were censored at the time of incident stroke.

The NAS was approved by the Institutional Review Boards of participating institutions and all participants provided written informed consent at each visit.

### Plasma extracellular miRNA profiling

Peripheral blood was collected in EDTA tubes and centrifuged at 1500 × *g* for 15 minutes to separate cell-free plasma, which was stored at −80°C. Total RNA was extracted from 1 mL of plasma using the Plasma/Serum Circulating and Exosomal RNA Purification Mini Kit (Slurry Format) (Norgen Biotek Corp., Ontario, Canada) [57]. RNA was serially eluted into 14 μL of RNAse-free water and stored at −80°C until sequencing. Prior to library prep, RNA concentration and size distribution were evaluated using an Agilent 2100 Bioanalyzer. Sequencing libraries were constructed using the NEBNext Small RNA Library Prep Set for Illumina (NEB, Ipswich, MA, USA) according to the manufacturer’s protocol with modifications. Reactions were conducted at one-fifth the recommended volume, 1.2 μL of each sample was used as input, the adapters were diluted to a 1:6 ratio, and 17 cycles of library amplification PCR were completed. DNA Clean and Concentrator Kits (Zymo Research, Irvine, CA, USA) were used to clean the library product of salts, proteins, and smaller nucleic acid fragments. Libraries were pooled (up to 144 samples) with equal volumes for each sample and the concentrations were quantified using the Quant-iT PicoGreen dsDNA Assay (Invitrogen, Waltham, MA, USA). The library size distribution was determined using a DNA HS Chip on a BioAnalyzer (Agilent Technologies, Santa Clara, CA, USA). The pools were size selected (115-150 base pairs [bp]) on a Pippin Prep instrument (Sage, Beverly, MA, USA) to remove adapter dimers and fragments larger than the target miRNA population and were sequenced to ~1,000,000 total reads per pool using a MiSeq instrument with a Nano flow cell (Illumina Inc, San Diego, CA, USA). This sequencing data was used to balance the samples into new pools for deeper sequencing on a HiSeq4000 instrument using single-end 75 bp runs.

Small RNA sequencing data were processed using a previously described workflow [58]. Data were mapped using the ExceRpt small RNA sequencing data analysis pipeline on the Genboree Workbench (http://genboree.org/site/exrna_toolset/). Mapping parameters specified a minimum read length of 15 nucleotides and 0 mismatches allowed, with the rest on default. We removed all samples with failed library preparation, defined as <10,000 total input reads (*n* = 56), and samples with low abundance of mapped miRNA reads (*n* = 9), defined as <100,000 total miRNA reads. In total, samples from 656 individuals passed quality control checks. Raw miRNA read counts were normalized via trimmed mean of M (TMM) method [59] and batch corrected using ComBat [60]. To increase our statistical power, extracellular miRNAs that were detectable in ≥ 70% of samples (381 miRNAs) were retained for all analyses.

### Cognitive assessments

Participants completed a battery of cognitive tests which included the Mini-Mental State Examination (MMSE). The MMSE is the most widely used screening instrument for cognitive decline and has been extensively validated and used both in clinical practice and epidemiological research as a dementia screening tool [61]. It assesses several cognitive domains such as orientation, immediate and short-term recall, attention and calculation, word finding, figure construction, reading and writing skills, and ability to follow a three-step command [62]. The range of scores in MMSE is 0–30; however, the maximum score in this study was 29 due to exclusion of the question on the county of residence because counties in Massachusetts are generally not known and thus are of little diagnostic utility [56]. This approach has been used previously to provide more robust results on this dataset [63, 64].

The present analysis includes cognitive data from study visits at or after the baseline visit, which we define as the visit where blood was drawn for the plasma miRNA measure, between 1996–2014. Participants completed up to 6 cognitive assessments over the study period (Table 2). Multiple imputation by chained equations was used to impute missing MMSE scores (and physical activity measures, used in sensitivity analyses) using the ‘mice’ package in R. The predictors used to impute missing data were the following: age (years), maximum education (years), first language (English/not English), computer experience (yes/no), physical activity (total metabolic equivalent hours/week), body mass index, hypertension (yes/no), alcohol intake (< 2 drinks/day, ≥ 2 drinks/day), smoking status (never, current/former), diabetes mellitus (yes/no), coronary heart disease (yes/no), stroke (yes/no), dark fish consumption (< once per week, ≥ once per week), MMSE score, and the scores of other cognitive tests, specifically the digit span backward test (total number and longest span of digits recalled), a verbal fluency task, a constructional praxis task (visual drawings: sum and weighted sum of drawings score), immediate and delayed recall of a 10-word list, and the mean and number correct in a pattern comparison test.

### Statistical analyses

The dataset was inspected for implausible and out-of-range values and one outlier with an MMSE score of 2 was removed (although including this observation in analyses did not change results). Descriptive statistics for study participants were calculated (mean and median for continuous variables and frequency for categorical variables). Differences between NAS participants included in the analyses (*N* = 530) and those excluded due to stroke (*N* = 48) were assessed using *t*-tests for continuous variables and chi-squared tests for categorical variables. The Mann-Whitney *U* test was applied for physical activity which exhibited a skewed distribution. These tests were two-sided with significance level 0.05. Descriptive statistics (minimum, maximum, mean, SD, and first/second/third quartiles) were calculated for each of the miRNAs that met the 70% detection threshold. Distributions of miRNA reads (normalized, batch-corrected counts per million reads) were examined by plotting histograms for each plasma miRNA, and Spearman correlations between plasma miRNAs were calculated. Reads (counts per million, normalized and batch-corrected) were log_2_ transformed for input in the analyses.

We first assessed the cross-sectional relationship between each of the detectable plasma miRNAs and MMSE score among men with MMSE measured at their baseline visit (*N* = 457, Figure 1 blue box) using linear regression models (Equation A). We then used linear mixed models with random intercepts for individual to assess the association between plasma miRNAs and longitudinal changes in cognition (*N* = 530, Figure 1 red box). Mixed models allow for differences in the number of repeated measures across participants; therefore, both persons with a single cognitive assessment and those with multiple assessments are included in our dataset and contribute to our primary estimates. The outcome for this analysis was MMSE score for subject *i* at visit *j*. The models included a linear term for time since the baseline assessment (i.e., follow up time) and an interaction term for plasma miRNA and follow up time; this cross-product represents the association between a given miRNA and rate of change in MMSE over time (Equation B).


$$\begin{array}{l} MMSE_{Baseline}=\beta_{0}+\beta_{1}\times miRNA+\beta_{2}\times Age+\beta_{3}\times Education\\ \;\;\;\;\;\;+\beta_{4}\times Alcohol+\beta_{5}\times SmokingStatus+\varepsilon \end{array}\text{(A)}$$

$$\begin{array}{l} MMSE_{ij}=\beta_{0i}+\beta_{1}\times miRNA_{i}+\beta_{2}\times Time_{fromBaseline}+\beta_{3}\\ \;\;\;\;(Time_{fromBaseline}\times miRNA_{i})+\beta_{4}\times Age_{i}+\beta_{5}\times Education_{i}\\ \;\;\;\;+\beta_{6}\times Alcochol_{i}+\beta_{7}\;SmokingStatus_{i}+\varepsilon_{ij} \end{array}\text{(B)}$$

All analyses were adjusted for predictors of cognitive function and potential confounders identified *a priori*, including baseline age (years), education (years), alcohol consumption (<2 or ≥2 drinks/day), and smoking status (never, current/former). We did not adjust for race/ethnicity because this sample was primarily non-Hispanic white (97%). Because variants in the apolipoprotein-E gene (*APOE*) are associated with risk of AD [65–67] and may influence cognition [68], we assessed the association between *APOE* polymorphisms and global cognitive function in this sample. Combinatorial and bivariate analyses of polymorphisms rs7412 and rs429358 did not show any association with MMSE, as also reported previously for this cohort [69], and therefore we did not adjust for allelic variants of *APOE*.

Analyses were performed separately for each plasma miRNA. We controlled for multiple comparisons using False Discovery Rates (FDR) and results were considered significant at FDRs < 0.05. We used R software (Version 4.0.5) for all statistical analyses [70].

### Sensitivity analyses

In sensitivity analyses, we additionally adjusted all models for diabetes (physician-diagnosed or having fasting glucose above 126 mg/dL), hypertension (yes/no), and measured or imputed physical activity (total metabolic equivalent hours/week). To control for practice effects [71], we adjusted for a variable indicating whether the data was from the participant’s first ever cognitive assessment. We also restricted the analyses to participants who were white (*N* = 443 for cross-sectional analysis, *N* = 513 for longitudinal analysis).

To adjust for potential selection bias due to loss to follow up, we constructed inverse probability of censoring weighted estimators using the baseline age, education, alcohol consumption, and smoking status as predictors and ran weighted linear mixed models among complete cases only (*N* = 526 with observed MMSE).

### Functional and pathway enrichment analyses

The biological relevance of the statistically significant plasma miRNAs identified by the linear regression models and linear mixed models was explored *a posteriori* via KEGG (Kyoto Encyclopedia of Genes and Genomes) pathway enrichment analysis performed using a miRNA pathway analysis web server, DIANA-miRPath v.3 (http://www.microrna.gr/miRPathv3) [72]. Fisher’s exact test was used to identify enriched pathways. Benjamini and Hochberg FDR-corrected *p*-values < 0.05 were considered statistically significant.

### Availability of data and materials

The data used in this study are subject to the rules and regulations of the U.S. Department of Veterans Affairs (VA) and are not publicly available. They may be made available upon reasonable request and compliance with the appropriate conditions as imposed by the VA.

## Supplementary Materials

Supplementary Figures

Supplementary Table 1

Supplementary Table 2

Supplementary Table 3

Supplementary Table 4

Supplementary Table 5

Supplementary Table 6

## References

[1] Jorm AF, Jolley D. The incidence of dementia: a meta-analysis. Neurology. 1998; 51:728–33. 10.1212/wnl.51.3.728 9748017

[2] Tucker-Drob EM. Neurocognitive functions and everyday functions change together in old age. Neuropsychology. 2011; 25:368–77. 10.1037/a0022348 21417532 PMC3086995

[3] Jekel K, Damian M, Wattmo C, Hausner L, Bullock R, Connelly PJ, Dubois B, Eriksdotter M, Ewers M, Graessel E, Kramberger MG, Law E, Mecocci P, et al. Mild cognitive impairment and deficits in instrumental activities of daily living: a systematic review. Alzheimers Res Ther. 2015; 7:17. 10.1186/s13195-015-0099-0 25815063 PMC4374414

[4] Park HL, O'Connell JE, Thomson RG. A systematic review of cognitive decline in the general elderly population. Int J Geriatr Psychiatry. 2003; 18:1121–34. 10.1002/gps.1023 14677145

[5] Tucker-Drob EM. Cognitive Aging and Dementia: A Life Span Perspective. Annu Rev Dev Psychol. 2019; 1:177–96. 10.1146/annurev-devpsych-121318-085204 34046638 PMC8153102

[6] Sadik N, Cruz L, Gurtner A, Rodosthenous RS, Dusoswa SA, Ziegler O, Van Solinge TS, Wei Z, Salvador-Garicano AM, Gyorgy B, Broekman M, Balaj L. Extracellular RNAs: A New Awareness of Old Perspectives. Methods Mol Biol. 2018; 1740:1–15. 10.1007/978-1-4939-7652-2_1 29388131 PMC6509047

[7] Tielking K, Fischer S, Preissner KT, Vajkoczy P, Xu R. Extracellular RNA in Central Nervous System Pathologies. Front Mol Neurosci. 2019; 12:254. 10.3389/fnmol.2019.00254 31680858 PMC6811659

[8] Quinn JF, Patel T, Wong D, Das S, Freedman JE, Laurent LC, Carter BS, Hochberg F, Van Keuren-Jensen K, Huentelman M, Spetzler R, Kalani MY, Arango J, et al. Extracellular RNAs: development as biomarkers of human disease. J Extracell Vesicles. 2015; 4:27495. 10.3402/jev.v4.27495 26320940 PMC4553262

[9] Wiedrick JT, Phillips JI, Lusardi TA, McFarland TJ, Lind B, Sandau US, Harrington CA, Lapidus JA, Galasko DR, Quinn JF, Saugstad JA. Validation of MicroRNA Biomarkers for Alzheimer's Disease in Human Cerebrospinal Fluid. J Alzheimers Dis. 2019; 67:875–91. 10.3233/JAD-180539 30689565 PMC6687305

[10] Yan Z, Zhou Z, Wu Q, Chen ZB, Koo EH, Zhong S. Presymptomatic Increase of an Extracellular RNA in Blood Plasma Associates with the Development of Alzheimer's Disease. Curr Biol. 2020; 30:1771–82.e3. 10.1016/j.cub.2020.02.084 32220323

[11] Sheinerman KS, Toledo JB, Tsivinsky VG, Irwin D, Grossman M, Weintraub D, Hurtig HI, Chen-Plotkin A, Wolk DA, McCluskey LF, Elman LB, Trojanowski JQ, Umansky SR. Circulating brain-enriched microRNAs as novel biomarkers for detection and differentiation of neurodegenerative diseases. Alzheimers Res Ther. 2017; 9:89. 10.1186/s13195-017-0316-0 29121998 PMC5679501

[12] Lugli G, Cohen AM, Bennett DA, Shah RC, Fields CJ, Hernandez AG, Smalheiser NR. Plasma Exosomal miRNAs in Persons with and without Alzheimer Disease: Altered Expression and Prospects for Biomarkers. PLoS One. 2015; 10:e0139233. 10.1371/journal.pone.0139233 26426747 PMC4591334

[13] Giau VV, Bagyinszky E, An SSA. Potential Fluid Biomarkers for the Diagnosis of Mild Cognitive Impairment. Int J Mol Sci. 2019; 20:4149. 10.3390/ijms20174149 31450692 PMC6747411

[14] De Felice B, Montanino C, Oliva M, Bonavita S, Di Onofrio V, Coppola C. MicroRNA Expression Signature in Mild Cognitive Impairment Due to Alzheimer's Disease. Mol Neurobiol. 2020; 57:4408–16. 10.1007/s12035-020-02029-7 32737762 PMC7515963

[15] Giuliani A, Gaetani S, Sorgentoni G, Agarbati S, Laggetta M, Matacchione G, Gobbi M, Rossi T, Galeazzi R, Piccinini G, Pelliccioni G, Bonfigli AR, Procopio AD, et al. Circulating Inflamma-miRs as Potential Biomarkers of Cognitive Impairment in Patients Affected by Alzheimer's Disease. Front Aging Neurosci. 2021; 13:647015. 10.3389/fnagi.2021.647015 33776746 PMC7990771

[16] Kenny A, McArdle H, Calero M, Rabano A, Madden SF, Adamson K, Forster R, Spain E, Prehn JHM, Henshall DC, Medina M, Jimenez-Mateos EM, Engel T. Elevated Plasma microRNA-206 Levels Predict Cognitive Decline and Progression to Dementia from Mild Cognitive Impairment. Biomolecules. 2019; 9:734. 10.3390/biom9110734 31766231 PMC6920950

[17] Cummings JL. Biomarkers in Alzheimer's disease drug development. Alzheimers Dement. 2011; 7:e13–44. 10.1016/j.jalz.2010.06.004 21550318

[18] Vlachos IS, Paraskevopoulou MD, Karagkouni D, Georgakilas G, Vergoulis T, Kanellos I, Anastasopoulos IL, Maniou S, Karathanou K, Kalfakakou D, Fevgas A, Dalamagas T, Hatzigeorgiou AG. DIANA-TarBase v7.0: indexing more than half a million experimentally supported miRNA:mRNA interactions. Nucleic Acids Res. 2015; 43:D153–9. 10.1093/nar/gku1215 25416803 PMC4383989

[19] Paraskevopoulou MD, Georgakilas G, Kostoulas N, Vlachos IS, Vergoulis T, Reczko M, Filippidis C, Dalamagas T, Hatzigeorgiou AG. DIANA-microT web server v5.0: service integration into miRNA functional analysis workflows. Nucleic Acids Res. 2013; 41:W169–73. 10.1093/nar/gkt393 23680784 PMC3692048

[20] Sahu MR, Mondal AC. The emerging role of Hippo signaling in neurodegeneration. J Neurosci Res. 2020; 98:796–814. 10.1002/jnr.24551 31705587

[21] Sahu MR, Mondal AC. Neuronal Hippo signaling: From development to diseases. Dev Neurobiol. 2021; 81:92–109. 10.1002/dneu.22796 33275833

[22] Sanphui P, Biswas SC. FoxO3a is activated and executes neuron death via Bim in response to β-amyloid. Cell Death Dis. 2013; 4:e625. 10.1038/cddis.2013.148 23661003 PMC3674357

[23] Swistowski A, Zhang Q, Orcholski ME, Crippen D, Vitelli C, Kurakin A, Bredesen DE. Novel mediators of amyloid precursor protein signaling. J Neurosci. 2009; 29:15703–12. 10.1523/JNEUROSCI.4351-09.2009 20016085 PMC2823633

[24] Tanaka H, Homma H, Fujita K, Kondo K, Yamada S, Jin X, Waragai M, Ohtomo G, Iwata A, Tagawa K, Atsuta N, Katsuno M, Tomita N, et al. YAP-dependent necrosis occurs in early stages of Alzheimer's disease and regulates mouse model pathology. Nat Commun. 2020; 11:507. 10.1038/s41467-020-14353-6 31980612 PMC6981281

[25] Irwin M, Tare M, Singh A, Puli OR, Gogia N, Riccetti M, Deshpande P, Kango-Singh M, Singh A. A Positive Feedback Loop of Hippo- and c-Jun-Amino-Terminal Kinase Signaling Pathways Regulates Amyloid-Beta-Mediated Neurodegeneration. Front Cell Dev Biol. 2020; 8:117. 10.3389/fcell.2020.00117 32232042 PMC7082232

[26] Kweon JH, Kim S, Lee SB. The cellular basis of dendrite pathology in neurodegenerative diseases. BMB Rep. 2017; 50:5–11. 10.5483/bmbrep.2017.50.1.131 27502014 PMC5319658

[27] Liu B, Sun LH, Huang YF, Guo LJ, Luo LS. Protein phosphatase 2ACα gene knock-out results in cortical atrophy through activating hippo cascade in neuronal progenitor cells. Int J Biochem Cell Biol. 2018; 95:53–62. 10.1016/j.biocel.2017.12.015 29274472

[28] Madole JW, Ritchie SJ, Cox SR, Buchanan CR, Hernández MV, Maniega SM, Wardlaw JM, Harris MA, Bastin ME, Deary IJ, Tucker-Drob EM. Aging-Sensitive Networks Within the Human Structural Connectome Are Implicated in Late-Life Cognitive Declines. Biol Psychiatry. 2021; 89:795–806. 10.1016/j.biopsych.2020.06.010 32828527 PMC7736316

[29] Kerrisk ME, Cingolani LA, Koleske AJ. ECM receptors in neuronal structure, synaptic plasticity, and behavior. Prog Brain Res. 2014; 214:101–31. 10.1016/B978-0-444-63486-3.00005-0 25410355 PMC4640673

[30] Doyle KP, Cekanaviciute E, Mamer LE, Buckwalter MS. TGFβ signaling in the brain increases with aging and signals to astrocytes and innate immune cells in the weeks after stroke. J Neuroinflammation. 2010; 7:62. 10.1186/1742-2094-7-62 20937129 PMC2958905

[31] Buckwalter MS, Wyss-Coray T. Modelling neuroinflammatory phenotypes in vivo. J Neuroinflammation. 2004; 1:10. 10.1186/1742-2094-1-10 15285805 PMC500895

[32] Tesseur I, Zou K, Esposito L, Bard F, Berber E, Can JV, Lin AH, Crews L, Tremblay P, Mathews P, Mucke L, Masliah E, Wyss-Coray T. Deficiency in neuronal TGF-beta signaling promotes neurodegeneration and Alzheimer's pathology. J Clin Invest. 2006; 116:3060–9. 10.1172/JCI27341 17080199 PMC1626127

[33] Wyss-Coray T. Transforming growth factor-beta signaling pathway as a therapeutic target in neurodegeneration. J Mol Neurosci. 2004; 24:149–53. 10.1385/JMN:24:1:149 15314264

[34] Wyss-Coray T. Tgf-Beta pathway as a potential target in neurodegeneration and Alzheimer's. Curr Alzheimer Res. 2006; 3:191–5. 10.2174/156720506777632916 16842094

[35] Tesseur I, Wyss-Coray T. A role for TGF-beta signaling in neurodegeneration: evidence from genetically engineered models. Curr Alzheimer Res. 2006; 3:505–13. 10.2174/156720506779025297 17168649

[36] Daulatzai MA. Cerebral hypoperfusion and glucose hypometabolism: Key pathophysiological modulators promote neurodegeneration, cognitive impairment, and Alzheimer's disease. J Neurosci Res. 2017; 95:943–72. 10.1002/jnr.23777 27350397

[37] Snowden SG, Ebshiana AA, Hye A, An Y, Pletnikova O, O'Brien R, Troncoso J, Legido-Quigley C, Thambisetty M. Association between fatty acid metabolism in the brain and Alzheimer disease neuropathology and cognitive performance: A nontargeted metabolomic study. PLoS Med. 2017; 14:e1002266. 10.1371/journal.pmed.1002266 28323825 PMC5360226

[38] Toledo JB, Arnold M, Kastenmüller G, Chang R, Baillie RA, Han X, Thambisetty M, Tenenbaum JD, Suhre K, Thompson JW, John-Williams LS, MahmoudianDehkordi S, Rotroff DM, et al, and Alzheimer's Disease Neuroimaging Initiative and the Alzheimer Disease Metabolomics Consortium. Metabolic network failures in Alzheimer's disease: A biochemical road map. Alzheimers Dement. 2017; 13:965–84. 10.1016/j.jalz.2017.01.020 28341160 PMC5866045

[39] Wang J, Wei R, Xie G, Arnold M, Kueider-Paisley A, Louie G, Mahmoudian Dehkordi S, Blach C, Baillie R, Han X, De Jager PL, Bennett DA, Kaddurah-Daouk R, Jia W. Peripheral serum metabolomic profiles inform central cognitive impairment. Sci Rep. 2020; 10:14059. 10.1038/s41598-020-70703-w 32820198 PMC7441317

[40] Vingtdeux V, Sergeant N, Buée L. Potential contribution of exosomes to the prion-like propagation of lesions in Alzheimer’s disease. Front Physiol. 2012; 3:229. 10.3389/fphys.2012.00229 22783199 PMC3389776

[41] Cheng L, Zhao W, Hill AF. Exosomes and their role in the intercellular trafficking of normal and disease associated prion proteins. Mol Aspects Med. 2018; 60:62–8. 10.1016/j.mam.2017.11.011 29196098

[42] Hartmann A, Muth C, Dabrowski O, Krasemann S, Glatzel M. Exosomes and the Prion Protein: More than One Truth. Front Neurosci. 2017; 11:194. 10.3389/fnins.2017.00194 28469550 PMC5395619

[43] Lusardi TA, Phillips JI, Wiedrick JT, Harrington CA, Lind B, Lapidus JA, Quinn JF, Saugstad JA. MicroRNAs in Human Cerebrospinal Fluid as Biomarkers for Alzheimer's Disease. J Alzheimers Dis. 2017; 55:1223–33. 10.3233/JAD-160835 27814298 PMC5587208

[44] Burgos K, Malenica I, Metpally R, Courtright A, Rakela B, Beach T, Shill H, Adler C, Sabbagh M, Villa S, Tembe W, Craig D, Van Keuren-Jensen K. Profiles of extracellular miRNA in cerebrospinal fluid and serum from patients with Alzheimer's and Parkinson's diseases correlate with disease status and features of pathology. PLoS One. 2014; 9:e94839. 10.1371/journal.pone.0094839 24797360 PMC4010405

[45] Nie C, Sun Y, Zhen H, Guo M, Ye J, Liu Z, Yang Y, Zhang X. Differential Expression of Plasma Exo-miRNA in Neurodegenerative Diseases by Next-Generation Sequencing. Front Neurosci. 2020; 14:438. 10.3389/fnins.2020.00438 32457573 PMC7227778

[46] Kumar P, Dezso Z, MacKenzie C, Oestreicher J, Agoulnik S, Byrne M, Bernier F, Yanagimachi M, Aoshima K, Oda Y. Circulating miRNA biomarkers for Alzheimer's disease. PLoS One. 2013; 8:e69807. 10.1371/journal.pone.0069807 23922807 PMC3726785

[47] Gibson EA, Goldsmith J, Kioumourtzoglou MA. Complex Mixtures, Complex Analyses: an Emphasis on Interpretable Results. Curr Environ Health Rep. 2019; 6:53–61. 10.1007/s40572-019-00229-5 31069725 PMC6693349

[48] Devenney E, Hodges JR. The Mini-Mental State Examination: pitfalls and limitations. Pract Neurol. 2017; 17:79–80. 10.1136/practneurol-2016-001520 27903765

[49] Abbott RA, Skirrow C, Jokisch M, Timmers M, Streffer J, van Nueten L, Krams M, Winkler A, Pundt N, Nathan PJ, Rock P, Cormack FK, Weimar C. Normative data from linear and nonlinear quantile regression in CANTAB: Cognition in mid-to-late life in an epidemiological sample. Alzheimers Dement (Amst). 2018; 11:36–44. 10.1016/j.dadm.2018.10.007 30623017 PMC6305838

[50] Piscopo P, Bellenghi M, Manzini V, Crestini A, Pontecorvi G, Corbo M, Ortona E, Carè A, Confaloni A. A Sex Perspective in Neurodegenerative Diseases: microRNAs as Possible Peripheral Biomarkers. Int J Mol Sci. 2021; 22:4423. 10.3390/ijms22094423 33922607 PMC8122918

[51] Mantione KJ, Kream RM, Kuzelova H, Ptacek R, Raboch J, Samuel JM, Stefano GB. Comparing bioinformatic gene expression profiling methods: microarray and RNA-Seq. Med Sci Monit Basic Res. 2014; 20:138–42. 25149683 10.12659/MSMBR.892101 PMC4152252

[52] Godoy PM, Barczak AJ, DeHoff P, Srinivasan S, Etheridge A, Galas D, Das S, Erle DJ, Laurent LC. Comparison of Reproducibility, Accuracy, Sensitivity, and Specificity of miRNA Quantification Platforms. Cell Rep. 2019; 29:4212–22.e5. 10.1016/j.celrep.2019.11.078 31851944 PMC7499898

[53] Wang Z, Gerstein M, Snyder M. RNA-Seq: a revolutionary tool for transcriptomics. Nat Rev Genet. 2009; 10:57–63. 10.1038/nrg2484 19015660 PMC2949280

[54] Bell B, Rose CL, Damon A. The Veterans Administration longitudinal study of healthy aging. Gerontologist. 1966; 6:179–84. 10.1093/geront/6.4.179 5342911

[55] Power MC, Weisskopf MG, Alexeeff SE, Coull BA, Spiro A 3rd, Schwartz J. Traffic-related air pollution and cognitive function in a cohort of older men. Environ Health Perspect. 2011; 119:682–7. 10.1289/ehp.1002767 21172758 PMC3094421

[56] Weisskopf MG, Wright RO, Schwartz J, Spiro A 3rd, Sparrow D, Aro A, Hu H. Cumulative lead exposure and prospective change in cognition among elderly men: the VA Normative Aging Study. Am J Epidemiol. 2004; 160:1184–93. 10.1093/aje/kwh333 15583371

[57] Alexander R, Laurent L. RNA isolation from human serum and plasma samples using the Norgen exosomal RNA purification mini kit. Protoc Exch. 2015. 10.1038/protex.2015.116

[58] Srinivasan S, Yeri A, Cheah PS, Chung A, Danielson K, De Hoff P, Filant J, Laurent CD, Laurent LD, Magee R, Moeller C, Murthy VL, Nejad P, et al. Small RNA Sequencing across Diverse Biofluids Identifies Optimal Methods for exRNA Isolation. Cell. 2019; 177:446–62.e16. 10.1016/j.cell.2019.03.024 30951671 PMC6557167

[59] Robinson MD, Oshlack A. A scaling normalization method for differential expression analysis of RNA-seq data. Genome Biol. 2010; 11:R25. 10.1186/gb-2010-11-3-r25 20196867 PMC2864565

[60] Johnson WE, Li C, Rabinovic A. Adjusting batch effects in microarray expression data using empirical Bayes methods. Biostatistics. 2007; 8:118–27. 10.1093/biostatistics/kxj037 16632515

[61] Tombaugh TN, McIntyre NJ. The mini-mental state examination: a comprehensive review. J Am Geriatr Soc. 1992; 40:922–35. 10.1111/j.1532-5415.1992.tb01992.x 1512391

[62] Folstein MF, Folstein SE, McHugh PR. "Mini-mental state". A practical method for grading the cognitive state of patients for the clinician. J Psychiatr Res. 1975; 12:189–98. 10.1016/0022-3956(75)90026-6 1202204

[63] Colicino E, Giuliano G, Power MC, Lepeule J, Wilker EH, Vokonas P, Brennan KJM, Fossati S, Hoxha M, Spiro A 3rd, Weisskopf MG, Schwartz J, Baccarelli AA. Long-term exposure to black carbon, cognition and single nucleotide polymorphisms in microRNA processing genes in older men. Environ Int. 2016; 88:86–93. 10.1016/j.envint.2015.12.014 26724585 PMC4755894

[64] Farooqui Z, Bakulski KM, Power MC, Weisskopf MG, Sparrow D, Spiro A 3rd, Vokonas PS, Nie LH, Hu H, Park SK. Associations of cumulative Pb exposure and longitudinal changes in Mini-Mental Status Exam scores, global cognition and domains of cognition: The VA Normative Aging Study. Environ Res. 2017; 152:102–8. 10.1016/j.envres.2016.10.007 27770710 PMC5135609

[65] Alzheimer’s Association. 2015 Alzheimer's disease facts and figures. Alzheimers Dement. 2015; 11:332–84. 10.1016/j.jalz.2015.02.003 25984581

[66] Raber J, Huang Y, Ashford JW. ApoE genotype accounts for the vast majority of AD risk and AD pathology. Neurobiol Aging. 2004; 25:641–50. 10.1016/j.neurobiolaging.2003.12.023 15172743

[67] Liu CC, Liu CC, Kanekiyo T, Xu H, Bu G. Apolipoprotein E and Alzheimer disease: risk, mechanisms and therapy. Nat Rev Neurol. 2013; 9:106–18. 10.1038/nrneurol.2012.263 23296339 PMC3726719

[68] O'Donoghue MC, Murphy SE, Zamboni G, Nobre AC, Mackay CE. APOE genotype and cognition in healthy individuals at risk of Alzheimer's disease: A review. Cortex. 2018; 104:103–23. 10.1016/j.cortex.2018.03.025 29800787

[69] Prada D, Colicino E, Power MC, Cox DG, Weisskopf MG, Hou L, Spiro Iii A, Vokonas P, Zhong J, Sanchez-Guerra M, Herrera LA, Schwartz J, Baccarelli AA. Influence of multiple APOE genetic variants on cognitive function in a cohort of older men - results from the Normative Aging Study. BMC Psychiatry. 2014; 14:223. 10.1186/s12888-014-0223-x 25085564 PMC4149270

[70] Dean CB, Nielsen JD. Generalized linear mixed models: a review and some extensions. Lifetime Data Anal. 2007; 13:497–512. 10.1007/s10985-007-9065-x 18000755

[71] Vivot A, Power MC, Glymour MM, Mayeda ER, Benitez A, Spiro A 3rd, Manly JJ, Proust-Lima C, Dufouil C, Gross AL. Jump, Hop, or Skip: Modeling Practice Effects in Studies of Determinants of Cognitive Change in Older Adults. Am J Epidemiol. 2016; 183:302–14. 10.1093/aje/kwv212 26825924 PMC4753282

[72] Vlachos IS, Zagganas K, Paraskevopoulou MD, Georgakilas G, Karagkouni D, Vergoulis T, Dalamagas T, Hatzigeorgiou AG. DIANA-miRPath v3.0: deciphering microRNA function with experimental support. Nucleic Acids Res. 2015; 43:W460–6. 10.1093/nar/gkv403 25977294 PMC4489228

