# Supplementary material for: Extracellular microRNA and cognitive function in a prospective cohort of older men: The Veterans Affairs Normative Aging Study
Source: Aging (Albany NY). 2022 Sep 6;14(17):6859–86. doi: 10.18632/aging.204268 (PMC9512498; doi:10.18632/aging.204268)
Supplement: Supplementary Figures [file aging-14-204268-s001.pdf]

SUPPLEMENTARY FIGURES

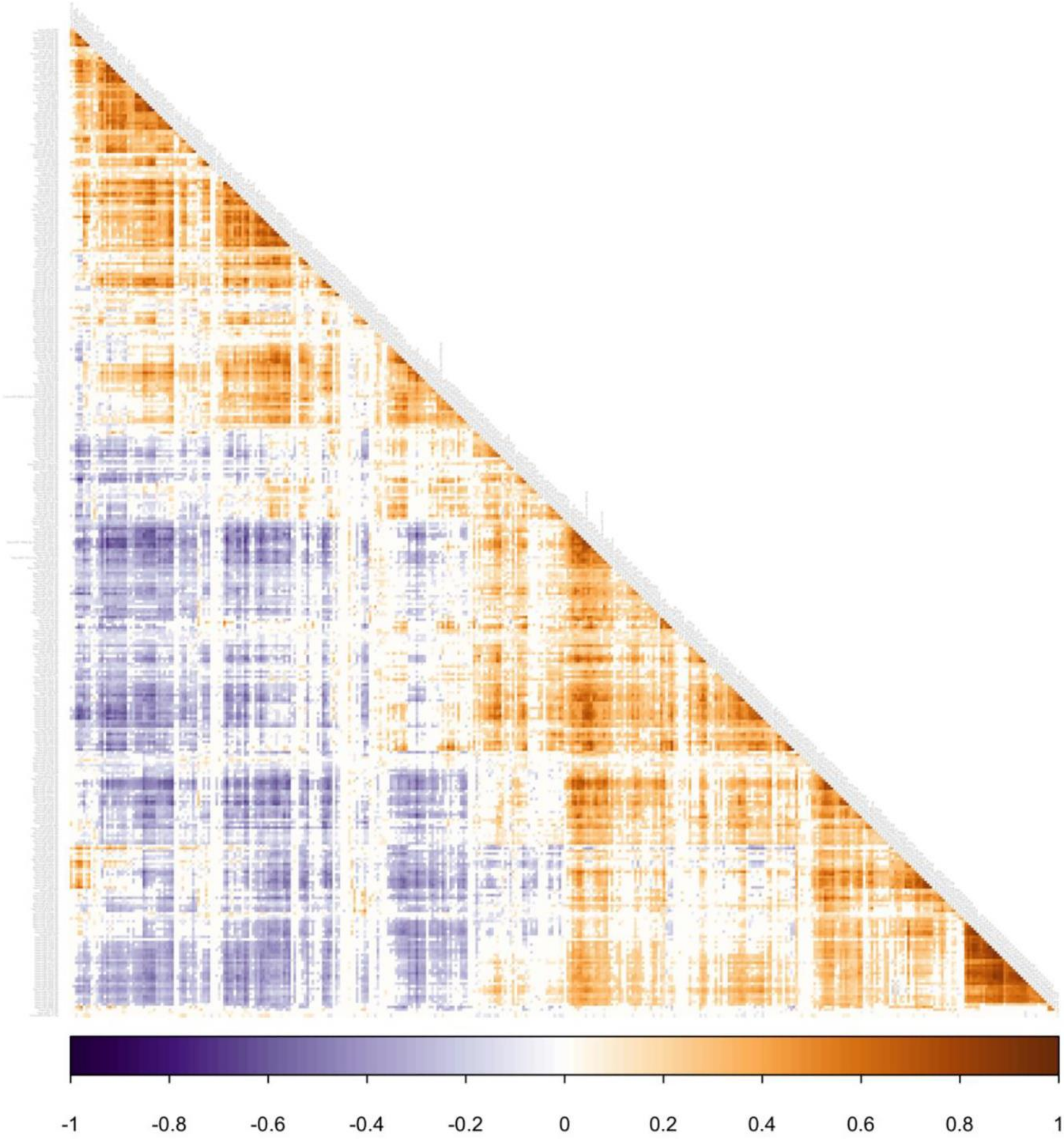

Supplementary Figure 1. Spearman correlation matrix of reads (normalized, batch-corrected counts per million) of the 381 plasma miRNAs detected in ≥ 70% of samples. MiRNAs are ordered by hierarchical clustering, and only correlations significant at $p < 0.05$ are shown.

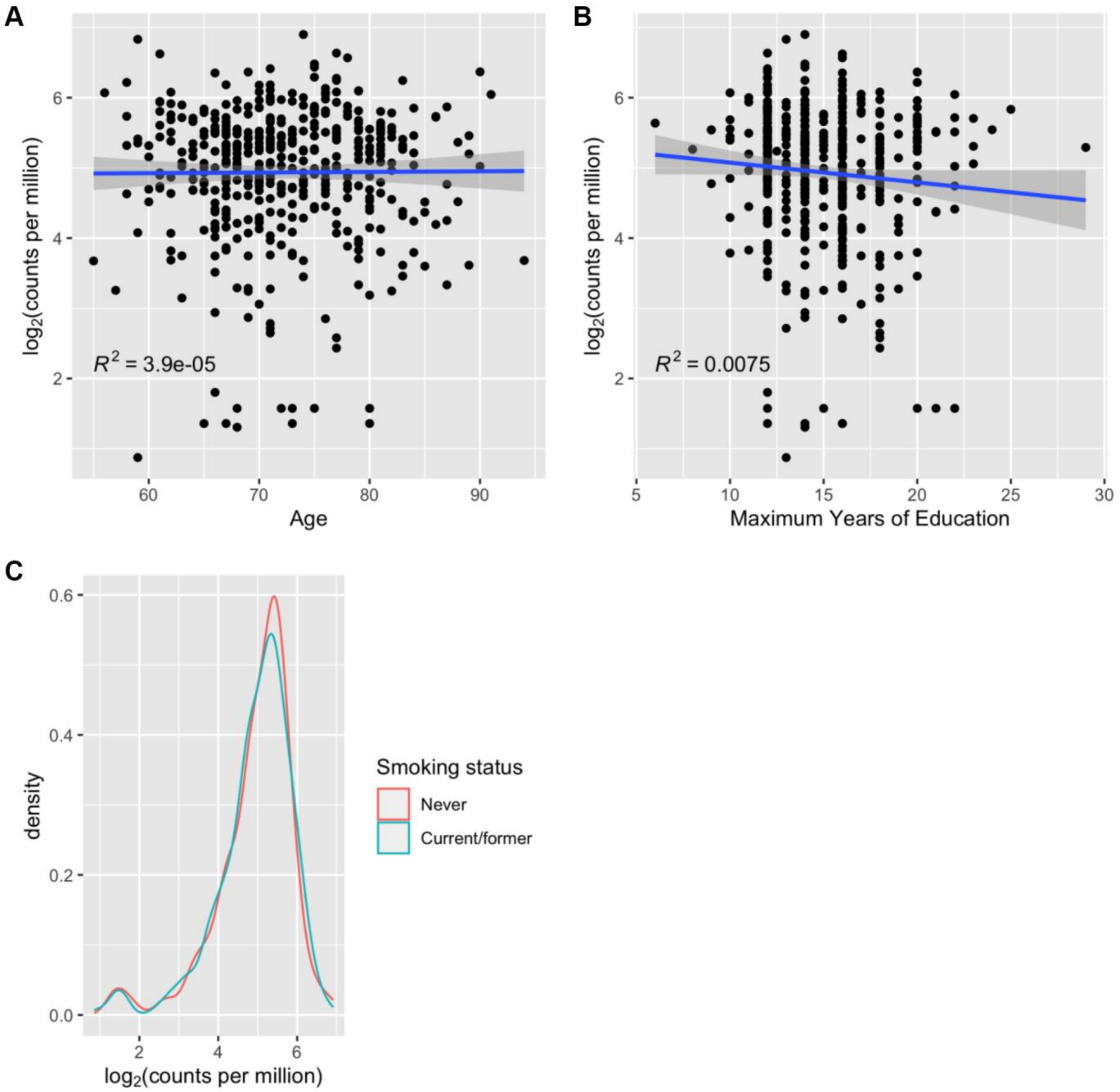

Supplementary Figure 2. Relationship between abundance (in $\log_2$ counts per million) of hsa-miR-148a-5p with cross-sectional age, education, and smoking status (*N* = 457). Correlation between hsa-miR-148a-5p ($\log_2$ counts per million) with age (**A**) and maximum years of education (**B**). The distribution of hsa-miR-148a-5p ($\log_2$ counts per million) is shown for current/former smokers and never smokers in panel (**C**).

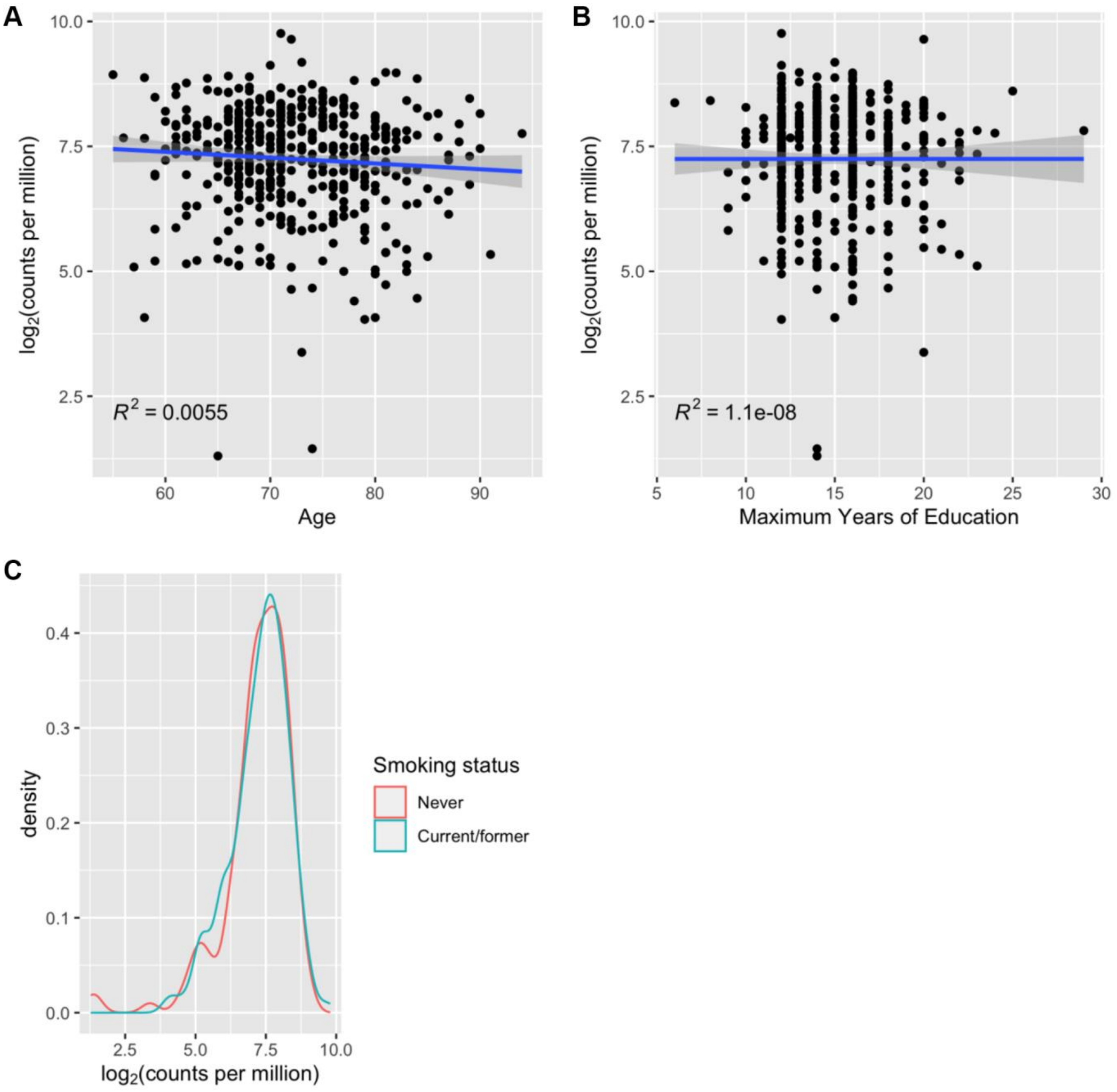

Supplementary Figure 3. Relationship between abundance (in log_2 counts per million) of hsa-miR-335-3p with cross-sectional age, education, and smoking status (*N* = 457). Correlation between hsa-miR-335-3p (log_2 counts per million) with age (**A**) and maximum years of education (**B**). The distribution of hsa-miR-335-3p (log_2 counts per million) is shown for current/former smokers and never smokers in panel (**C**).

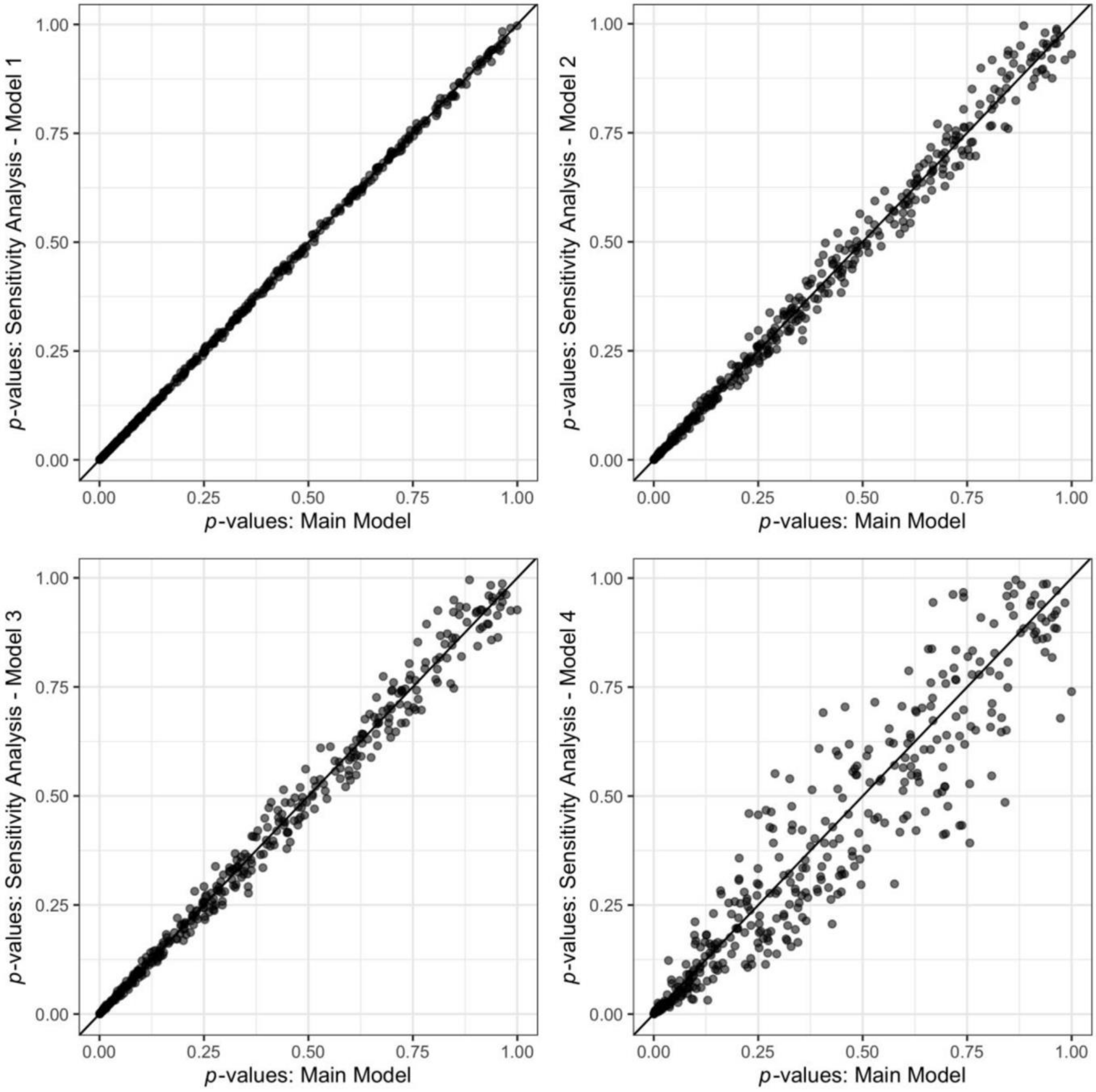

Supplementary Figure 4. QQplots displaying unadjusted *p*-values of models used in sensitivity analyses vs. the Main Model (linear model adjusted for age, education, alcohol consumption, and smoking status). Model 1: Main model additionally adjusted for an indicator for whether the cognitive assessment was the participant's first-ever time undergoing cognitive testing; Model 2: Main model further adjusted for physical activity, diabetes and hypertension status; Model 3: Model 2 additionally adjusting for the first cognitive test indicator; Model 4: Main model restricted to white participants only (N = 443).

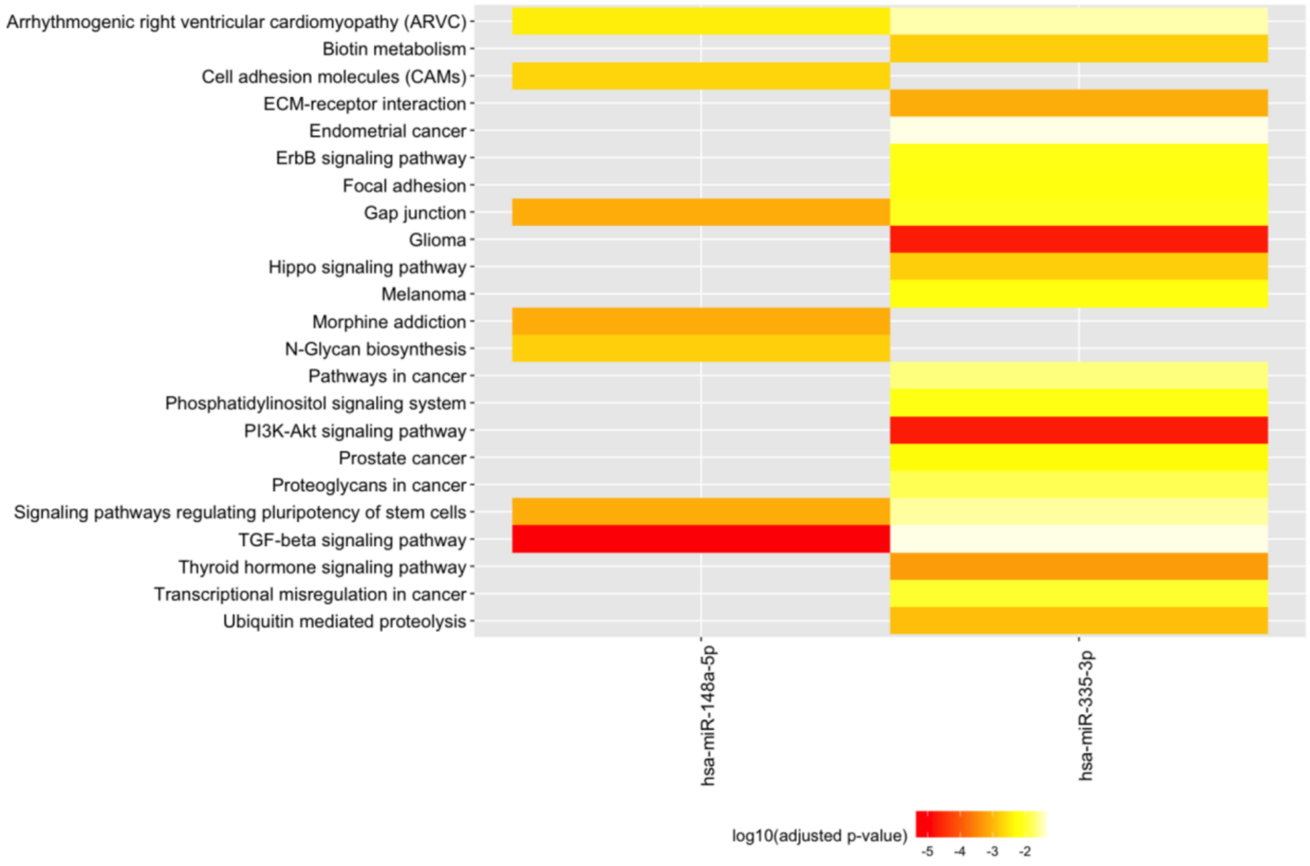

Supplementary Figure 5. KEGG pathway enrichment analysis of predicted target genes of hsa-miR-148a-5p and hsa-miR-335-3p. KEGG pathway analysis of predicted miRNA target genes for hsa-miR-148a-5p (625 genes) and hsa-miR-335-3p (2,984 genes) identified by microT-CDS v.5.

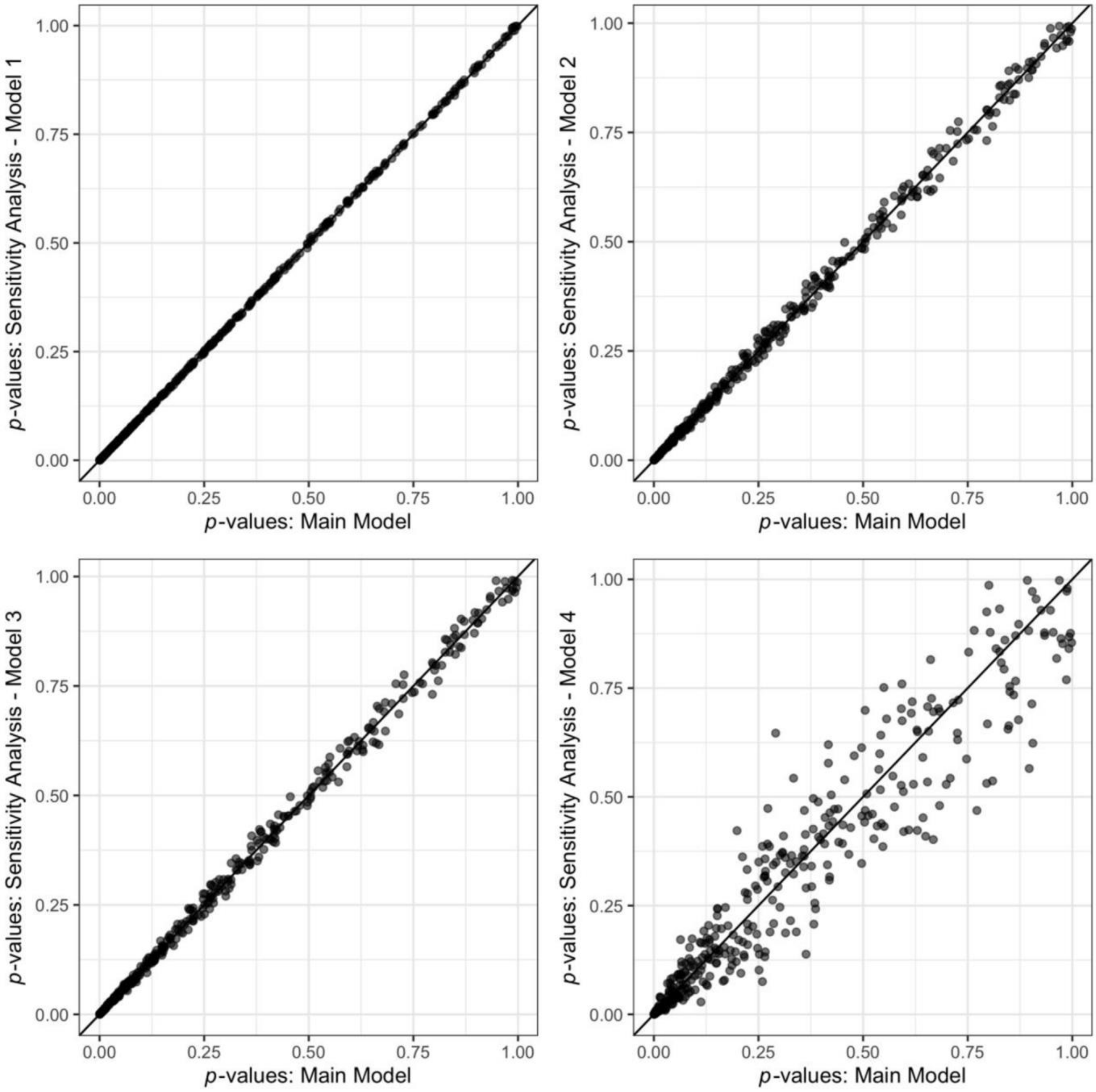

Supplementary Figure 6. QQplots displaying unadjusted *p*-values of models used in sensitivity analyses vs. the Main Model (linear mixed model adjusted for age, education, alcohol consumption, smoking status, follow up time, and included an interaction term for miRNA and follow up time). Model 1: Main model additionally adjusted for an indicator for whether the cognitive assessment was the participant's first-ever time undergoing cognitive testing; Model 2: Main model further adjusted for physical activity, diabetes and hypertension status; Model 3: Model 2 additionally adjusting for the first cognitive test indicator; Model 4: Main model restricted to white participants only (*N* = 513).

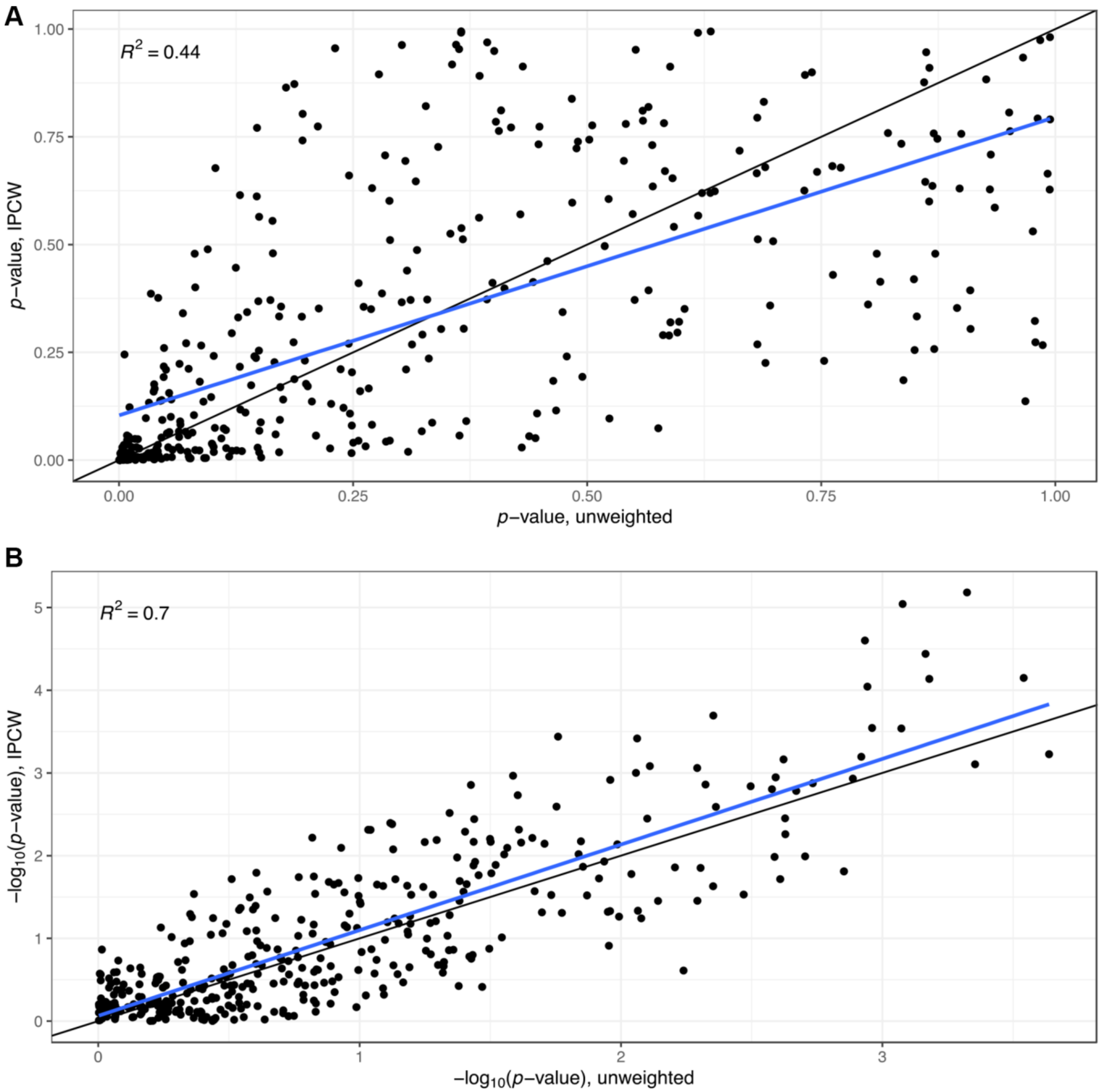

Supplementary Figure 7. QQplot Displaying Unadjusted (**A**) *p*-values and (**B**) -$\log_{10}$(*p*-values) of Unweighted Complete Case Analysis vs. Inverse Probability of Censoring-Weighted (IPCW) Complete Case Analysis. These linear mixed models were adjusted for age, education, alcohol consumption, smoking status, follow up time, and included an interaction term for miRNA and follow up time. N = 526 subjects with 1,285 person-visits. Black line: slope = 1. Blue line: linear regression line.
