# Supplementary material for: Extracellular microRNA and cognitive function in a prospective cohort of older men: The Veterans Affairs Normative Aging Study
Source: Aging (Albany NY). 2022 Sep 6;14(17):6859–86. doi: 10.18632/aging.204268 (PMC9512498; doi:10.18632/aging.204268)
Supplement: Supplementary Table 6 [file aging-14-204268-s007.pdf]

Supplementary Table 6. Baseline characteristics of included (*N* = 530) and excluded (*N* = 48) Normative Aging Study (NAS) participants.

	Stroke = No	Stroke = Yes[†]
	(*N* = 530)	(*N* = 48)
**Age (years)[*]**		
Mean (SD)	72.0 (6.8)	77.2 (7.5)
Median (Min, Max)	71.0 (55.0, 94.0)	77.0 (57.0, 92.0)
**Race/Ethnicity**		
White	513 (96.8%)	47 (97.9%)
Black	12 (2.3%)	0 (0%)
Hispanic	4 (0.8%)	1 (2.1%)
Missing	1 (0.2%)	0 (0%)
**Max Education (years)**		
Mean (SD)	14.9 (2.9)	15.3 (3.4)
Median (Min, Max)	14.0 (6.0, 29.0)	15.0 (11.0, 30.0)
**Alcohol Consumption (drinks/day)**		
< 2	438 (82.6%)	40 (83.3%)
≥ 2	92 (17.4%)	8 (16.7%)
**Smoking Status**		
Never	158 (29.8%)	19 (39.6%)
Current/Former	372 (70.2%)	29 (60.4%)
**Physical Activity (MET-hrs/week)**		
Mean (SD)	14.3 (17.8)	12.1 (12.4)
Median (Min, Max)	7.75 (0.25, 138.17)	7.58 (0.25, 43.6)
1st quartile, 3rd quartile	3.09, 19.62	3.12, 16.02
Missing	29 (5.5%)	3 (6.3%)
**Diabetes**		
No	435 (82.1%)	34 (70.8%)
Yes	95 (17.9%)	14 (29.2%)
**Hypertension**		
No	151 (28.5%)	11 (22.9%)
Yes	379 (71.5%)	37 (77.1%)

These participants had at least one cognitive assessment on or after the baseline visit, defined as the visit when blood was drawn for plasma miRNA analysis. Abbreviations: SD: Standard deviation; MET: Metabolic equivalent of task, measured in hours (hrs) per week. One MET is defined as the energy expenditure for sitting quietly, which, for the average adult, approximates 3.5 mL of oxygen uptake per kilogram of body weight per minute (1.2 kcal/min for a 70-kg individual). [†]These participants were excluded from analyses. [*]$p < 0.0001$
